# The Role of Dopamine in *Drosophila* Larval Classical Olfactory Conditioning

**DOI:** 10.1371/journal.pone.0005897

**Published:** 2009-06-12

**Authors:** Mareike Selcho, Dennis Pauls, Kyung-An Han, Reinhard F. Stocker, Andreas S. Thum

**Affiliations:** 1 Department of Biology, University of Fribourg, Fribourg, Switzerland; 2 Department of Biology and The Huck Institute Neuroscience and Genetics Graduate Program, Pennsylvania State University, University Park, Pennsylvania, United States of America; Centre de Recherches su la Cognition Animale - Centre National de la Recherche Scientifique and Université Paul Sabatier, France

## Abstract

Learning and memory is not an attribute of higher animals. Even *Drosophila* larvae are able to form and recall an association of a given odor with an aversive or appetitive gustatory reinforcer. As the *Drosophila* larva has turned into a particularly simple model for studying odor processing, a detailed neuronal and functional map of the olfactory pathway is available up to the third order neurons in the mushroom bodies. At this point, a convergence of olfactory processing and gustatory reinforcement is suggested to underlie associative memory formation. The dopaminergic system was shown to be involved in mammalian and insect olfactory conditioning. To analyze the anatomy and function of the larval dopaminergic system, we first characterize dopaminergic neurons immunohistochemically up to the single cell level and subsequent test for the effects of distortions in the dopamine system upon aversive (odor-salt) as well as appetitive (odor-sugar) associative learning. Single cell analysis suggests that dopaminergic neurons do not directly connect gustatory input in the larval suboesophageal ganglion to olfactory information in the mushroom bodies. However, a number of dopaminergic neurons innervate different regions of the brain, including protocerebra, mushroom bodies and suboesophageal ganglion. We found that dopamine receptors are highly enriched in the mushroom bodies and that aversive and appetitive olfactory learning is strongly impaired in dopamine receptor mutants. Genetically interfering with dopaminergic signaling supports this finding, although our data do not exclude on naïve odor and sugar preferences of the larvae. Our data suggest that dopaminergic neurons provide input to different brain regions including protocerebra, suboesophageal ganglion and mushroom bodies by more than one route. We therefore propose that different types of dopaminergic neurons might be involved in different types of signaling necessary for aversive and appetitive olfactory memory formation respectively, or for the retrieval of these memory traces. Future studies of the dopaminergic system need to take into account such cellular dissociations in function in order to be meaningful.

## Introduction

*Drosophila* larvae learn to avoid an odor (the conditioned stimulus [CS]) that was paired with salt (aversive unconditioned stimulus [US]). Conversely, if the same CS is paired with sugar (appetitive US), larvae develop a preference toward it. Thus, depending on previous experience, the same CS can trigger either avoidance or preference [1], [2]. How are these antagonistic behaviors modulated on the cellular and molecular level?

The olfactory pathway of the larva has been described in detail [2]. Twenty-one olfactory receptor neurons (ORNs) are assembled in the dorsal organ, the unique larval olfactory organ [3]–[5]. ORNs usually express one, occasionally two ligand-binding odorant receptors, defining the range of odors to which they respond. Each of the 21 ORNs targets one among 21 glomeruli in the larval antennal lobe (al) [4], [5]. Second-order olfactory projection neurons (PNs) connect the al with higher order olfactory centers, the lateral horn and the mushroom body (mb) calyx [5]–[7]. In adult flies, the lateral horn seems to be involved in innate odor recognition [8]–[10], whereas for the adult and larval mbs there is strong evidence for being a center for olfactory learning [2], [11], [12]; [but see also 13, reporting a contribution of the mbs in innate odor preferences]. In contrast to the olfactory CS, which is mediated via the PNs, punishment or reward signals were suggested to reach the mbs via separate, yet largely unknown pathways [14]. Accordingly, the simultaneous arrival of the CS and the US at the mbs would strengthen the synapses from the intrinsic mb Kenyon cells to output neurons.

The gustatory system of the larva is less well described than the olfactory system. A majority of the estimated 90 larval gustatory receptor neurons (GRNs) [15] are located in three external sense organs, terminal, dorsal and ventral organ, and three pharyngeal organs [3], [16], [17]. Other putative taste organs may occur in thoracic and abdominal segments [18], [19]. As shown for adult flies, GRNs either respond to high or low salt concentrations, sugar or bitter substances [20]. Salt was reported to be mediated by ionic channels that are encoded by the *pickpocket* (*ppk*) gene family [21], [22], whereas sweet and bitter compounds bind to members of a family of 7-transmembrane gustatory receptors [23]–[27]. The GRN afferents of the larval head chemosensory organs project via four different nerves to the suboesophageal ganglion (sog) [3], [15]. So far, in *Drosophila*, no second-order gustatory neurons are described that would be suited to pass on gustatory stimuli to the mbs [but see for the honeybee 28], [29].

For *Apis mellifera*, *Gryllus bimaculatus* and *Drosophila melanogaster*, there is evidence that the two biogenic amines dopamine (DA) and octopamine (OA) are specifically involved in punishment and reward signaling, respectively [17], [30]–[33]. Furthermore, for *Drosophila* larvae, activation of DA neurons and concurrent application of an odor was shown to be sufficient to induce aversive memory. Whereas paired activation of tyraminergic (TA), the precursor of OA, and OA neurons together with application of the same odor was sufficient to elicit appetitive memory [34]. Recently, it was also shown that blocking DA neuron output during training, but not during test, specifically impairs aversive memory. On the other hand, output of TA/OA neurons is necessary during training for appetitive memory [33]. Together, these data suggest distinct, conserved mechanisms for punishment and reward processing among insects. However, the idea of DA being exclusively involved in punishment signaling was challenged by a recent study in adult *Drosophila*, which suggested that the expression of dDA1 is a necessary prerequisite for both aversive and appetitive olfactory learning [35].

In *Drosophila*, the enzyme tyrosine hydroxylase (TH, *CG10118*) catalyzes the rate-limiting step of DA biosynthesis [36] and is specifically expressed in all dopaminergic cells. By using antibodies raised against TH [36]–[39], the larval DA system was shown to consist of two clusters per hemisphere comprising four to ten neurons each and a stereotyped pattern of three to five paired (lateral) or unpaired (medial) neurons per segment in the sog and ventral nerve cord (vnc) [36]. Postsynaptically, two G-protein coupled DA receptors were described in *Drosophila*, called *dDA1* (*Drosophila dopamine receptor 1*; *CG9652*) and *DAMB (dopamine receptor in mushroom bodies*; *CG18741)*. Both show increased expression levels in the mbs [35], [40], [41] and both were reported to be capable of mediating a DA-induced increase in cyclic adenosine monophosphate (cAMP) levels [40]–[44]. This is of considerable interest as the cAMP cascade is known to be one of the core signal transduction pathways for elementary forms of short-term and long-term memory [reviewed in 45]. Correspondingly, dDA1 is required locally in the adult mbs in order to form olfactory associations, suggesting that output of DA neurons onto the mbs is necessary for learning [35]. Still, our knowledge about the organization of the DA system and its possible function in aversive and appetitive classical conditioning in insects is limited and in particular lacks single-cell resolution.

To overcome this limitation, sophisticated methods of genetic manipulation can be applied. Using the GAL4/UAS system [46]–[49], almost any gene of choice can reproducibly be expressed in a defined set of cells. For example, the temperature-sensitive dominant negative *shibire^ts1^* (*shi^ts1^*) can be used as an effector gene to interfere with neurotransmission. It encodes temperature-sensitive dynamin GTPase that disrupts synaptic vesicle recycling at temperatures above 30°C [50], [51]. Due to its conditional activity, *shi^ts1^* can be used to interfere with neurotransmission specifically during the time of the learning experiment, excluding developmental phenotypes. In order to anatomically untangle neuronal circuits at the single-cell level, the flp-out system [52], a modification of the traditional GAL4/UAS system, can be applied. It allows random labeling of single cells from the ensemble of cells visualized by the GAL4 driver line.

Here we use a bipartite approach to analyze the DA system in the central nervous system (cns) of the *Drosophila* larva with respect to classical olfactory conditioning. First, we study by immunohistochemistry the input and output regions of DA neurons as well as the expression patterns of the DA receptors dDA1 and DAMB. We describe the anatomy of single DA neurons for the first time. Finally, we analyze the function of the DA system by applying a paradigm for classical larval olfactory learning [1], [53]–[55] to larvae defective in DA signaling or mutant for a DA receptor. In contrast to a previous report [33], our data suggest, when considered in their entirety, that DA is involved in sensing or processing of olfactory and gustatory stimuli apart from a role in both aversive and appetitive larval olfactory learning. For the latter case, the involvement of DA is implicated especially from the DA receptor data. The discrepancy between the previous report [33] and our own evidence might be explained by differences in the training protocols (see discussion). Given the diverse classes of DA neurons we have described on the single-cell level, we anticipate that a dissociation in function with respect to aversive and appetitive learning may be observed only when taking note of the individual type of neuron – if at all.

## Results

### Nomenclature of the Larval Brain Regions

**Figure 1:**
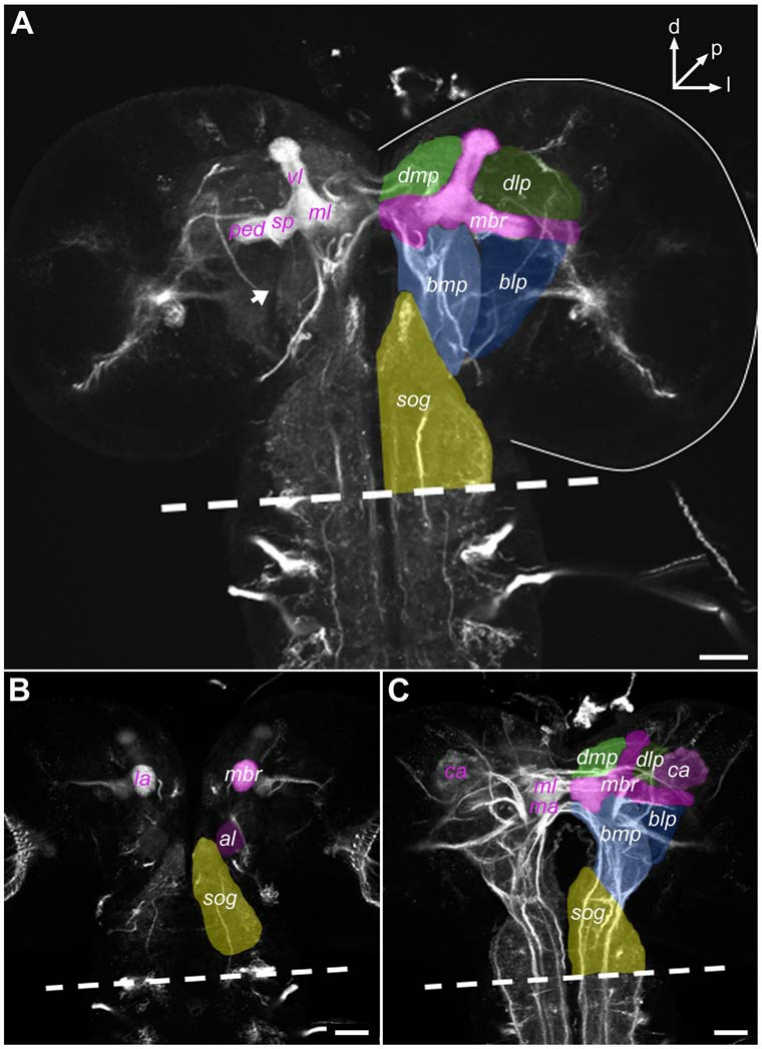
Nomenclature of Larval Brain Regions. For mapping neurons of the DA system, we defined brain subregions [see 57]. The orientation refers to the body axis (A: d: dorsal; l: lateral; p: posterior). Preparations were flattened during mounting and thus eliminate the typical 90° rotation of the central nervous system (cns) at the intersection between the suboesophageal ganglion (sog) and ventral nerve cord (dashed line). The background was stained by a combination of anti-FasII (for axon tracts) and anti-ChAT (for neuropiles). (A) shows the brain at a middle anteroposterior level; (B) and (C) represent more anterior and more posterior levels, respectively. Separated by the mushroom body region (mbr), each hemisphere was divided in four subregions: dorsomedial protocerebrum (dmp), dorsolateral protocerebrum (dlp), basomedial protocerebrum (bmp) and basolateral protocerebrum (blp). bmp and blp are separated by a lack of the anti-ChAT staining (A arrow). The mb nomenclature is depicted in the right hemisphere (A–C): vertical lobe (vl), medial lobe (ml), spur (sp), pedunculus (ped), calyx (ca) medial appendix (ma) and lateral appendix (la). Antennal lobes (al) in the anterior part of the brain (B) and the mb in the posterior part (C) were taken as additional subregions. Scale bars: 50 µm.

To analyze the cellular anatomy of the DA system in the larval cns, we used anti-Fasciclin II (FasII)/anti-Cholineacetyltransferase (ChAT) background staining (Figure 1), which label axonal tracts [56] and neuropiles [15], respectively. As we focused exclusively on the larva, we used stage-independent abbreviations (i.e., antennal lobe rather than larval antennal lobe). To ease comparison with the adult brain, our nomenclature is based on the body-axis of the larva. Also, our terminology does not reflect the flattening of the cns during mounting and therefore ignores its 90° rotation near the intersection between sog and vnc (dashed line in Figure 1). To locate the different types of neurons and their processes in the brain, we divided each hemisphere into four subregions. Simplifying the nomenclature of Younossi-Hartenstein and coworkers [57], we called them dorsomedial protocerebrum, dorsolateral protocerebrum, basomedial protocerebrum and basolateral protocerebrum separated by the mb region (Figure 1A). In addition, we considered the al in the anterior part of the brain and the mb calyx in its posterior part as additional subregions (Figure 1B and 1C). For the mb we used the following nomenclature from medial to lateral: medial lobe, vertical lobe [58] (called “dorsal lobe” in some insects), spur, pedunculus and calyx (Figure 1). Finally, for the larval-specific “bulbous outswellings” [59] or “axonal side branches” [60] which occur exclusively at the lateral and medial end of the medial lobe, and were often mistaken with its adjoining spur, we introduced the terms “lateral appendix” and “medial appendix”.

### Basic Anatomy of the Dopaminergic System in the Larval CNS

**Figure 2:**
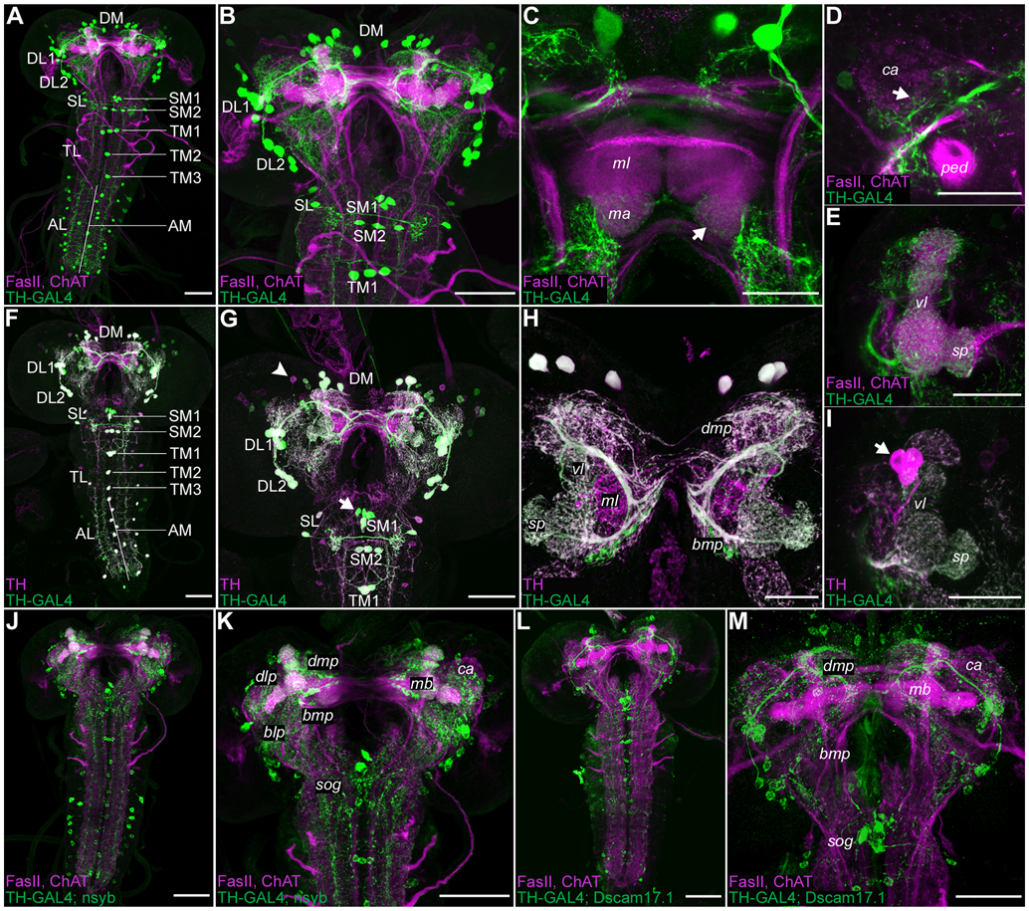
Anatomy of the Dopaminergic System in the Larval CNS Based on the TH-GAL4 Driver and anti-TH Staining. (A–E) TH-GAL4/UAS-*Cameleon2.1* expressing cells (green) are shown on combined anti-FasciclinII (FasII)/anti-Cholineacetyltransferase (ChAT) background staining (magenta). (F–I) Relation of tyrosine hydroxylase (TH) immunoreactivity (magenta) and TH-GAL4 expression (green). Presynaptic (J and K) and postsynaptic (L and M) regions of TH-GAL4 expressing cells, labeled by UAS-*nsyb::GFP* (nsyb) and UAS-*Dscam[17.1]::GFP* (Dscam17.1), respectively (green), as shown on anti-FasII/anti-ChAT background (magenta). All panels represent projections of confocal sections, except D which shows a single optical section. (A and B) TH-GAL4-positive cell clusters. (C) TH-GAL4 expressing neurons innervate the medial appendices (ma; arrow) but not the medial lobes (ml) of the mushroom bodies (mbs); (D) they arborize in the lateral mb calyx (ca; arrow), (E) in the vertical lobe (vl), spur (sp) and lateral appendix of the mb. (F and G) TH-immunoreactivity overlaps with TH-GAL4 expression in most of the neurons. However, a few cell bodies are TH-positive but do not express TH-GAL4 (arrowhead), while others are only labeled by TH-GAL4 (arrow). (H) The mls are not innervated by TH-GAL4 expressing neurons, but by anti-TH-positive neurons whose cell bodies are shown in I (arrow). (J and K) Presynaptic structures of TH-GAL4 expressing neurons are spread all-over the larval cns, such as the mbs, dorsal and ventral protocerebra and sog. (L and M) Postsynaptic structures are less dense in the mbs, but occupy the dorsomedial (dmp), dorsolateral protocerebra (dlp) and basal structures of the brain apart from the thoracic and abdominal neuromeres. Scale bars: A,B,F,G,J–M 50 µm; D,E,H,I 25 µm.

**Figure 3:**
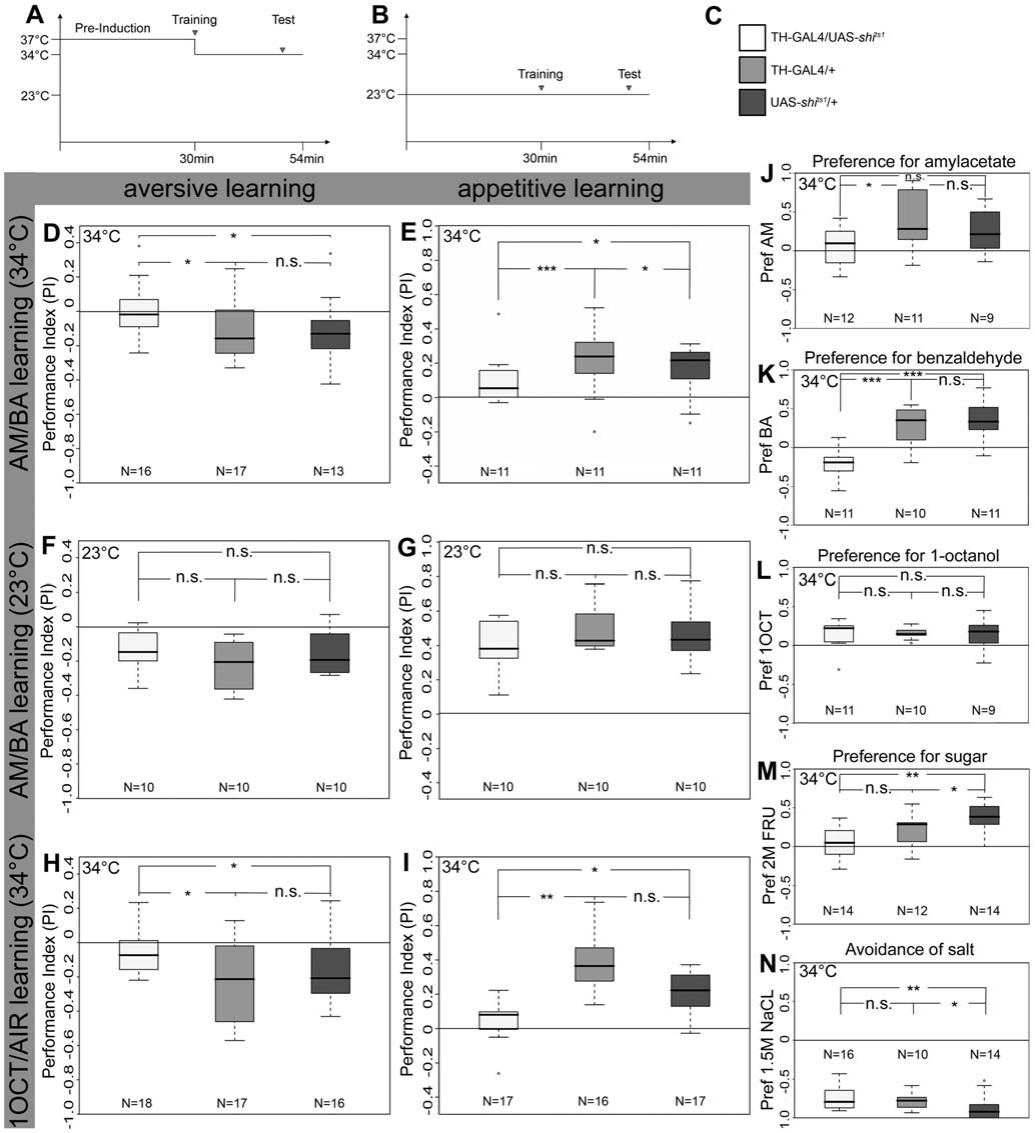
The Role of Dopaminergic Neurons in Larval Aversive and Appetitive Olfactory Learning. Protocols for training and testing larvae at restrictive and permissive temperature are shown in (A) and (B), respectively. (C) The shading chosen for experimental and control animals applies to the entire figure. (D) TH-GAL4/UAS-*shi^ts1^* larvae did not show any significant performance in aversive olfactory conditioning at restrictive temperature, neither in two-odor learning assays [using amylacetate (AM) against benzaldehyde (BA)] (p = 0.057), nor in single-odor assays [using 1-octanol (1OCT) against air] (H, p = 0.495). In both cases larvae of the control genotypes TH-GAL4/+ and UAS-*shi^ts1^* showed significantly higher performances. For appetitive AM/BA learning, performance of experimental larvae was not different from chance level (p = 0.174) and was strongly reduced compared to TH-GAL4/+ and UAS-*shi^ts1^*/+ (E, p = 5.67×10^−6^ and p = 0.015). For appetitive 1OCT/AIR learning, performance was over chance level (p = 0.008), but was reduced compared to the control larvae (I; p = 0.003 compared to TH-GAL4/+ and p = 0.035 compared to UAS-*shi^ts1^*/+). At permissive temperature, larvae of all genotypes performed at wild type levels, for both aversive (F) and appetitive olfactory conditioning (G). (J) TH-GAL4/UAS-*shi^ts1^* larvae did not show any significant preference for AM (p = 0.569) in contrast to TH-GAL4/+ (p = 0.01). (K) Surprisingly, they strongly avoided BA, which was attractive for larvae of both controls. (L) For 1OCT, larvae of all genotypes showed wild type levels of preference. (M) Although experimental larvae were not significantly different in their ability to perceive sugar than TH-GAL4/+ larvae (p = 0.116), they did not perform significantly over chance level (p = 0.462). We note that the performance of UAS-*shi^ts1^*/+ larvae was significantly different from the response levels of both TH-GAL4/+ (p = 0.044) and experimental larvae (p = 0.001). (N) TH-GAL4/UAS-*shi^ts1^* larvae showed strong avoidance to salt which was not significantly different from avoidance in TH-GAL4/+ (p = 0.937). However, the particularly strong repulsion of UAS-*shi^ts1^* differed significantly from the other two lines.

**Figure 4:**
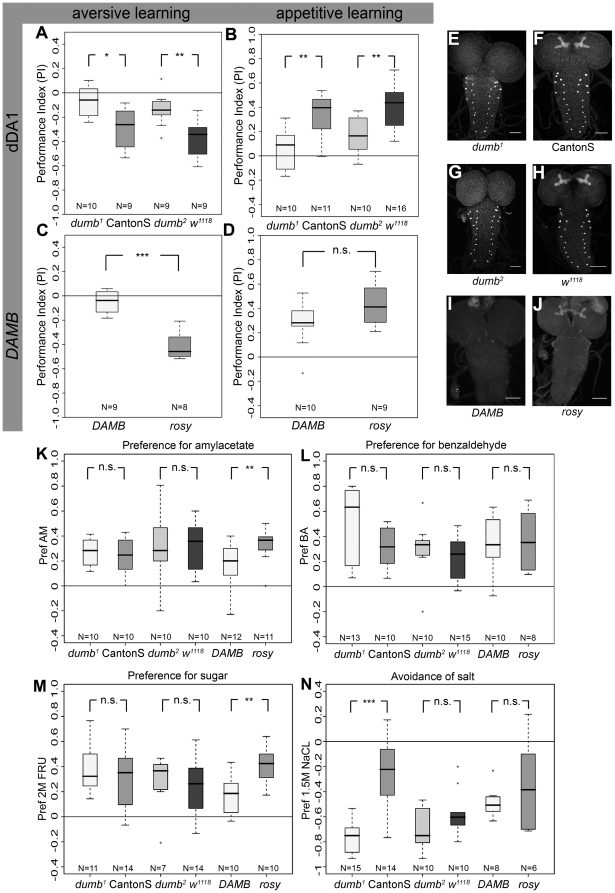
The Role of Dopamine Receptors in Larval Aversive and Appetitive Olfactory Learning. (A) For aversive learning, both *dumb^1^* and *dumb^2^* mutants performed significantly less than the corresponding control larvae CantonS and *w^1118^* (p = 0.013 for *dumb^1^* and CantonS; p = 0.004 for *dumb^2^* and *w^1118^*). Whereas learning scores of *dumb^1^* were not different from zero (p = 0.16), *dumb^2^* mutants performed still above chance level (p = 0.019). (B) *dumb^1^* mutants showed strongly reduced appetitive learning (p = 0.234 ; p = 0.006 for *dumb^1^* and CantonS). *dumb^2^* mutants performed above chance level (p = 0.009), but their scores were significantly reduced compared to control larvae *w^1118^* (p = 0.004). (C) Compared to *rosy* controls, *DAMB* mutants showed a strong reduction in aversive learning (p = 8.27×10^−5^), which was not different from zero (p = 0.203). (D) In appetitive learning, *DAMB* mutants were not different from control larvae (p = 0.112). (E–H) Staining with anti-dDA1 antibody in the larval central nervous system showed a strongly reduced expression of dDA1 in *dumb^1^* and *dumb^2^* mutants compared to CantonS and *w^1118^* controls. This difference was particularly visible in the mbs, but not in neurons situated in the ventral nerve cord (I,J) Staining with anti-DAMB antibody showed strongly reduced DAMB expression in *DAMB* mutant larvae compared to *rosy* controls. (K) Odor preferences for AM of *dumb^1^* (p = 0.676) and *dumb^2^* (p = 0.879) mutant larvae were not different from their controls. *DAMB* mutant larvae were significantly reduced in their AM preference (p = 0.005). (L) Odor preferences for BA of *dumb^1^* (p = 0.076), *dumb^2^* (p = 0.469) and DAMB (p = 0.858) mutant larvae were not different from their controls. (M) Neither *dumb^1^* (p = 0.661) nor *dumb^2^* (p = 0.411) showed a reduced naïve sugar preference, which was the case for *DAMB* (p = 0.002). (N) Neither *dumb^2^* (p = 0.140) nor *DAMB* (p = 0.651) showed a reduced naïve salt preference, which was the case for *dumb^1^* (p = 4.9×10^−5^).

Approximately 70 putative DA neurons have been described in the cns of third instar larvae by catecholamine histofluorescence [37] (see also Table 1) and by immunoreactivity to DA, TH [38] and Dopa decarboxylase (DDC) [39], [61], [62]. Apart from three bilaterally symmetrical clusters of DA neurons in the brain called DL1, DL2 and DM [63], DA cell bodies were reported from the sog and the thoracic and abdominal neuromeres (Table 1) [36]. For analyzing the gross anatomy of the larval DA system with respect to the published data [36]–[39], [61], [62], we used the TH-GAL4 driver line [36] to express either UAS*-mCD8::GFP* (data not shown) [60] or UAS*-Cameleon2.1* [64]. A significantly stronger signal was obtained with UAS*-Cameleon2.1* compared to UAS*-mCD8::GFP*, the former providing two anti-GFP binding sites, it allowed us to identify cells with low GAL4 expression levels. By double-labeling with anti-GFP and anti-TH antibodies we were able to visualize the DL1, DL2 and DM clusters in TH-GAL4 (Figure 2). In DL1, seven to eight cell bodies were labeled (Table 1), although the TH-GAL4 line labeled one neuron that was not TH-positive. DL2 consisted of about six cell bodies per hemisphere in TH-GAL4 (Table 1), all of which were TH-positive. In the DM cluster, only eight DA cells were strongly labeled in all brains; in the remaining cell bodies, staining intensity varied, depending on the applied effectors and antibodies (Table 1, Figure 2H and data not shown). Concerning the sog, previous studies categorized the DA neurons as paired and unpaired types [37], [38]. Based on our single cell labelings we doubt such a distinction. Rather we prefer the more neutral terms lateral and medial, describing exclusively the position of the cell body. The same nomenclature was applied for thoracic and abdominal neuromeres. In the sog we were able to distinguish two anteriomedial clusters, SM1 and SM2, and a more lateral cluster SL (Figure 2; Table 1). TH-GAL4 labeled about four cells in SM1 (Table 1) but only one of them was labeled by the anti-TH antibody, suggesting additional non-DA expression in three neurons. The SM2 cluster contained approximately three cells, which were all TH-positive (Table 1). The SL cluster of TH-GAL4 comprised about five cells per side; only three of them were double labeled and are therefore TH-positive. However, TH-GAL4 did not label three additional TH-positive cells (Table 1). Details about thoracic and abdominal DA clusters are provided in Table 1 and Figure 2. Taken together, TH-GAL4 labels a comprehensive set of DA neurons in the larval DA system and was therefore used in our behavioral approach (Figure 3). The expression pattern, however, is not complete and also includes a few TH-negative neurons [see also 36]. We next analyzed the cellular anatomy of TH-GAL4-positive cells in the larval brain. Due to their widespread arborization patterns, the anatomy of single DA neurons was difficult to untangle. Essentially, TH-GAL4 positive neurons innervated the protocerebra, the mbs, the sog as well as thoracic and abdominal ganglia (Figure 2). However, in insects DA is not only used as a neurotransmitter, but also as a neuromodulator [reviewed in 63], [65]–[67]. For example, Greer and colleagues have shown that the vesicular monoamine transporter mediates the transport of DA into secretory vesicles [68]. Therefore, if DA acts as a neuromodulator, these types of neurons would not make direct synaptic connections and show diffuse anatomical projections. Yet, due to the limited resolution of the confocal microscope, our data did not allow to distinguish between these possibilities. The al was weakly labeled by TH-GAL4 driven UAS-*Cameleon2.1*, but not by the anti-TH antibody (Figure S2). Therefore, it is unlikely, although not formally excluded, that the al is innervated by DA neurons. Focusing on the mbs, we noticed that the TH-GAL4 driven Cameleon2.1 did not reveal any innervation of the main branch of its medial lobes (Figure 2C). In contrast, the larval-specific medial and lateral appendices (see above), as well as the vertical lobes, the spurs and the calyces were all innervated (Figure 2B–2E). Interestingly, about four DA neurons per hemisphere, having their cell bodies anterior to the dorsal part of the vertical lobe, densely innervated the medial lobe, as shown by anti-TH staining (Figure 2I). Therefore, the main branch of the medial lobes is innervated by DA neurons that are not included in the TH-GAL4 expression pattern. We further analysed the DA system by expressing post- and presynaptic effectors via TH-GAL4, reflecting potential input and output regions of the DA neurons respectively. From the available postsynaptic effectors, UAS-*RDL::HA* (*resistence to dieldrin*) [69] preferentially accumulated in the cell bodies, whereas UAS-*PAK::GFP (p21/rac1-activated kinase)* [70] and UAS-*S97-DLG::GFP (Discs large)* [71] labeled the whole neuron including axons (for all effectors data not shown). Thus, our data were limited to the dendrite-specific *Drosophila Down Syndrome Cell Adhesion Molecule* conjugated to GFP (*Dscam[17.1]::GFP*) [72]. Similar to the adult fly [73], TH-GAL4/UAS-*Dscam[17.1]::GFP* larvae showed reduced staining in various parts of the brain including the mbs and sog compared to Cameleon2.1 (Figure 2L and 2M). In contrast, innervation was detected in the lateral horns, in the dorso- and basomedial protocerebra (Figure 2L and 2M) as well as in thoracic and abdominal neuromeres. To test the potential output regions of the DA system, we expressed – via the TH-GAL4 line – the presynaptic reporter genes *n-synaptobrevin::GFP*, *synaptotagmin::HA* and *synaptotagmin::GFP* [74], [75] which yielded similar results (data not shown). Most brain regions, as well as the sog, were labeled with the same intensity, suggesting that there are no spatially separated cellular outputs in these regions (Figure 2J and 2K). In contrast, the mbs showed a defined dense innervation at the vertical lobes, the spurs and the pedunculi (Figure 2J and 2K) suggesting that these mb regions are presynaptic sites of the DA system. Analyzing the expression patterns of two DA receptors dDA1 and DAMB further supported this interpretation (Figure 4E–4J). Both dDA1 and DAMB showed strong expression in the mb lobes and the pedunculi, apart from some expression in the vnc (Figure 4F, 4H and 4J). Nevertheless, due to the limitations of our immunohistochemical approach, we could not exclude low level receptor expression in other brain areas, as suggested by the presynaptic reporter expression (Figure 2J and 2K). To test whether the larval DA system is indeed involved in aversive and/or appetitive olfactory learning, we performed a set of conditioning experiments.

**Table 1:** Cell numbers of potential dopaminergic neurons in the larval central nervous system.

Neuropile (literature)	Literature	TH-GAL4; UAS-CAM2.1 anti-GFP	TH-GAL4; UAS-CAM2.1 anti-TH	Overlay of anti-GFP and anti-TH	Neuropile (this study)
**DL1 left**	∼7 (8)	7.6±0.2 (9)	7.6±0.3 (9)	**6.7±0.2 (9)**	**DL1 left**
**DL1 right**	∼6 (5)	7.1±0.3 (9)	7.3±0.2 (9)	**6.7±0.2 (9)**	**DL1 right**
**DL2 left**	∼7 (8)	5.7±0.2 (9)	5.7±0.2 (9)	**5.7±0.2 (9)**	**DL2 left**
**DL2 right**	∼6 (8)	6.0±0.2 (9)	6.0±0.2 (9)	**6.0±0.2 (9)**	**DL2 right**
**DM left**	∼6 (8)	35.2±3.6 (9)	40.2±3.4 (9)	**26.3±3.7 (9)**	**DM**
**DM right**	∼6 (8)				
**Sb Th unpaired**	∼4 (13)	4.3±0.4 (8)	1.0±0.0 (8)	**1.0±0.0 (8)**	**SM1**
		3.0±0.3 (8)	2.9±0.1 (8)	**2.8±0.2 (8)**	**SM2**
		3.0±0.0 (9)	3.0±0.0 (9)	**3.0±0.0 (9)**	**TM1**
		1.0±0.0 (9)	1.0±0.0 (9)	**1.0±0.0 (9)**	**TM2**
		1.0±0.0 (9)	1.0±0.0 (9)	**1.0±0.0 (9)**	**TM3**
**Sb Th paired**	∼4 (13)	5.3±1.0 (8)	6.1±0.5 (8)	**2.9±0.2 (8)**	**SL left**
		4.6±0.9 (8)	6.1±0.3 (8)	**3.0±0.3 (8)**	**SL right**
**TH lateral**	∼4 (13)	1.1±0.4 (8)	3.6±0.2 (8)	**0.3±0.2 (8)**	**TL left**
		1.3±0.5 (8)	4.0±0.0 (8)	**0.6±0.3 (8)**	**TL right**
**Ab unpaired**	∼7 (13)	10.3±0.8 (7)	9.1±0.1 (7)	**9.1±0.1 (7)**	**AM**
**Ab lateral**	∼14 (13)	23.0±1.4 (7)	13.9±0.1 (7)	**14.1±0.3 (7)**	**AL**
**Brain**	∼38	78.9±6.0 (8)	82.0±4.3 (8)	**61.5±4.4 (8)**	**Brain and S clusters**
**Sb and Th**	∼12	41.8±2.0 (6)	35.5±0.3 (6)	**29.2±0.5 (6)**	**T and A clusters**
**Ab**	∼19				
**Total**	∼69	120.2±7.3 (6)	119.3±3.9 (6)	**90.7±5.0 (6)**	**Total**

### Dopaminergic Neurons in Aversive and Appetitive Larval Olfactory Learning

We utilized a two-group, reciprocal training design [reviewed in 2]: to the first group of about 30 larvae an odor A is presented together with a gustatory US; next, these larvae are confronted with an odor B without US. Another group of larvae receives reciprocal training, i.e. odor A is presented without and odor B with reinforcement. Subsequently, the two groups are tested for their preference between A versus B. For aversive learning larvae were tested on a salt plate, for appetitive learning larvae were tested on a pure plate [1]. Relatively lower/higher preferences for A after punishing/rewarding A and B after punishing/rewarding B then reflect associative learning. Note that the association is measured as a performance of groups of larvae and not at the individual level. It was reported that larvae can associate an odor with a gustatory reward but do not recall the memory in a pleasant test situation (e.g. on a sugar plate for sugar learning) [1]. We therefore prefer to use the term “performance index” rather than “learning index”, as we measure a behavioral output during test that depends, at least in part, on the current test situation, which might not reflect the complete memory formed by the larva. In order to interfere with DA neurotransmission, we expressed several effectors via TH-GAL4, which covers more than 75% of the DA neurons in the larval brain (Table 1 and see above). Expressing tetanus toxin light chain [76], [77] did not result in a detectable mutant phenotype; moreover, expressing the inwardly rectifying potassium channel Kir2.1 [77], [78] yielded a strong developmental phenotype, respectively (data not shown). We thus chose UAS-*shi^ts1^* [50], [51] for blocking synaptic transmission. Expressing this effector in all ORNs by incubating Or83b-GAL4/UAS-*shi^ts1^* larvae [79] for 30 min at 37°C fully blocked odor preferences for both odors, whereas GAL4/+ and UAS/+ controls showed responses over chance level (Figure S1). Therefore, our restrictive temperature protocol for olfactory learning consisted of a 30 min pre-incubation at 37°C, followed by three 2.5 min training cycles and a 5 min test period, all at 34°C (Figure 3A). Note that this protocol was different to the one used in a recent study [33]. When the odor preference was tested at 31°C without pre-incubation, only a partial impairment of the naïve odor response was detected (data not shown). After conditioning the two odors amylacetate (AM) and benzaldehyde (BA) with salt as an aversive US, TH-GAL4/UAS-*shi^ts1^* larvae tested at restrictive temperature showed significantly reduced performance compared to both GAL4/+ and UAS/+ controls (p = 0.048 for TH-GAL4/+ and p = 0.031 for UAS-*shi^ts1^*/+). Moreover, the performance index of TH-GAL4/UAS-*shi^ts1^* was not different from zero (p = 0.057; Figure 3D). The memory impairment was specific to the restrictive temperature, as TH-GAL4/UAS-*shi^ts1^* larvae at permissive temperature showed similar performance as both control genotypes (p = 0.190 for TH-GAL4/+ and p = 0.496 for UAS-*shi^ts1^/+*; Figure 3F). Similarly, for appetitive olfactory conditioning using sugar as an US, scores for TH-GAL4/UAS-*shi^ts1^* experimental larvae were significantly reduced compared to both controls at restrictive temperature (p = 5.67×10^−6^ for TH-GAL4/+ and p = 0.015 for UAS-*shi^ts1^*/+). Again, their performance index was not different from zero (p = 0.174; Figure 3E). None of the three genotypes showed a reduction in performance at permissive temperature (p = 0.165 for TH-GAL4/+ and p = 0.578 for UAS-*shi^ts1^/+*; Figure 3G). In order to verify that any of the memory impairments observed upon *shibire^ts1^*-dependent block of synaptic transmission were due to impaired odor perception, we presented these cues to naïve larvae under the same conditions as for the associative assays. When AM was tested against air at restrictive temperature, naïve TH-GAL4/UAS-*shi^ts1^* larvae showed no reduction in the preference index compared to the UAS-*shi^ts1^*/+ control group (p = 0.213), but this index was not significantly different from zero (p = 0.569; Figure 3J). In addition, TH-GAL4/UAS-*shi^ts1^* larvae avoided BA when tested against air, whereas GAL4/+ and UAS/+ control groups were attracted by this compound (Figure 3K). Therefore, we cannot exclude that the reduced performance indices for aversive and appetitive learning of TH-GAL4/UAS-*shi^ts1^* larvae at restrictive temperature might be partially due to changes in the naïve responses, at least to BA. For a second set of conditioning experiments, we chose 1-octanol (1OCT) in a single odor learning assay (B. Gerber, Würzburg, personal communication), as the naïve odor response of TH-GAL4/UAS-*shi^ts1^* larvae to 1OCT was not significantly different at restrictive temperature compared to GAL4/+ (p = 0.756) and UAS/+ (p = 0.823) control larvae (Figure 3L). In this single odor assay again a two-group, reciprocal training design was utilized. For the first group of about 30 larvae, 1OCT was paired with a gustatory US and “no odor” without US. Another group of about 30 larvae received reciprocal training, i.e., 1OCT presented without and “no odor” with reinforcement; all other parameters were kept constant.

Again, TH-GAL4/UAS-*shi^ts1^* larvae tested at restrictive temperature showed significantly reduced performance after aversive olfactory conditioning compared to both GAL4/+ and UAS/+ controls in the single odor assay (Figure 3H; p = 0.047 for TH-GAL4/+ and p = 0.026 for UAS-*shi^ts1^*/+). For appetitive olfactory conditioning as well, scores for TH-GAL4/UAS-*shi^ts1^* larvae were significantly reduced at the restrictive temperature compared to the two controls (Figure 3I; p = 0.003 for TH-GAL4/+ and p = 0.035 for UAS-*shi^ts1^*/+); for aversive but not for appetitive conditioning the performance indices of experimental TH-GAL4/UAS-*shi^ts1^* larvae were not different from zero (p = 0.495).

Finally, we tested if the memory impairments observed upon *shibire^ts1^*-dependent block of synaptic transmission were not simply due to impaired salt or sugar perception. When naïve salt preference was tested at restrictive temperature, scores of TH-GAL4/UAS-*shi^ts1^* larvae were not reduced compared to the GAL4/+ control group (Figure 3N; p = 0.937). Consequently, the reduced performance of experimental larvae in the aversive single odor assay (Figure 3H) demonstrates that DA is required for aversive olfactory learning. When naïve sugar preference was tested at restrictive temperature, TH-GAL4/UAS-*shi^ts1^* larvae performed similarly as the GAL4/+ control group (p = 0.116). On the other hand, they showed a significant reduction compared to UAS-*shi^ts1^*/+ (p = 0.0011) and their sugar preference was not significantly different from zero (Figure 3M; p = 0.462). These results contrasted a recent report [33]. Taken together, we cannot exclude that the reduction in appetitive learning is due, at least partially, to changes in the naïve sugar response. To address the question whether DA is in fact required for appetitive olfactory learning we interfered with postsynaptic signaling by using DA receptor mutants.

### Dopamine Receptors in Aversive and Appetitive Larval Olfactory Learning

The five known subtypes of DA receptors belong to two main classes: D1-like receptors (with the subtypes D1 and D5) activate adenylyl cyclases through interactions via G_s_, whereas D2-like receptors (comprising D2, D3 and D4 subtypes) inhibit adenylyl cyclases and other effector molecules by interacting with G_i_/G_o_ [for reviews see 80]–[83]. In *Drosophila*, the *rutabaga* adenylyl cyclase, which is activated by G_s_, was shown to be required for olfactory learning [84]–[86]. We therefore focused on the *Drosophila* D1-like receptors dDA1 and DAMB (see below) [35], [40]. Two mutants called *dumb^1^* and *dumb^2^* were published for a D1-like DA receptor dDA1 [35]. *dumb^1^* – an inversion *[In(3LR)234]* with breakpoints at 67D and 88A [87] – was outcrossed several times by CantonS, which can therefore be used as an appropriate control. *dumb^2^* contains a piggyBac insertion [88] in the first intron in the dDA1 locus *[f02676]* and was backcrossed to *w^1118^*, serving as an appropriate control. If stained with a dDA1 antibody [41], both mutants showed a strongly reduced expression in the mbs of the larval brain (Figure 4E and 4G) compared to their controls (Figure 4F and 4H). For our learning experiments we used the AM/BA two odor olfactory learning assay. After aversive olfactory conditioning, *dumb^1^* mutant larvae had significantly reduced scores compared to CantonS wild-type controls (Figure 4A; p = 0.013). Similarly, *dumb^2^* mutants performed significantly lower than *w^1118^* control larvae (Figure 4A; p = 0.004). When appetitive olfactory conditioning was tested, both *dumb^1^* and *dumb^2^* mutant larvae also showed significantly reduced scores compared to the controls (Figure 4B; p = 0.006 for *dumb1* and CantonS and p = 0.004 for *dumb^2^* and *w^1118^*). For aversive and appetitive olfactory conditioning, the performance indices of *dumb^1^* mutant larvae were not significantly different from zero (p = 0.160 for aversive conditioning and p = 0.234 for appetitive conditioning), whereas the indices of *dumb^2^* mutants despite being strongly impaired after both types of conditioning were different from zero (Figure 4A and 4B; p = 0.019 for aversive conditioning and p = 0.010 for appetitive conditioning). This difference in performance may be due to a low endogenous expression of dDA1 in the *dumb^2^* mutant, as piggyBac is inserted in the first intron of the dDA1 locus leaving the second exon with its 5′ untranslated sequence and the start codon intact [35]. A second D1-like DA receptor in *Drosophila* is called *DAMB* [40]. We analyzed the behavior of a deletion strain in the *rosy* background that uncovers the *DAMB* gene and 5′ a second gene *CG1907*, a potential malate transporter. If stained with an anti-DAMB antibody [40], *DAMB* mutant larvae showed a strongly reduced expression in the larval brain (Figure 4I) compared to the *rosy* control (Figure 4J). For aversive olfactory conditioning, the performance of *DAMB* mutant larvae was reduced compared to *rosy* controls (p = 8.27×10^−5^); their scores were not different from zero (Figure 4C; p = 0.203). In contrast, appetitively conditioned *DAMB* mutant larvae did not perform significantly different from *rosy* controls (Figure 4D; p = 0.112). Therefore it is tempting to speculate that *DAMB* may be specifically involved in aversive olfactory learning. Nevertheless, the results have to be interpreted with care, as the deletion line is not specific for *DAMB* and also covers *CG1907*. To verify that the memory impairments observed by manipulation of DA receptor function were not simply due to impaired odor, salt or sugar perception, we performed control experiments in which these cues were presented under the same conditions as for the associative assays. Figure 4K–4N shows the performance for each genotype tested with respect to naïve olfactory behavior, salt avoidance and sugar attraction. For *dumb^2^* mutant larvae neither sugar perception (p = 0.411), nor salt avoidance (p = 0.140), AM preference (p = 0.879) or BA preference (P = 0.469) were significantly altered compared to *w^1118^* control larvae. Thus, memory impairment of the *dumb^2^* mutant larvae was attributable to impairment of acquisition and/or retrieval, rather than to changes in sensory perception. For *dumb^1^* mutant larvae neither sugar perception (p = 0.661) nor AM preference (p = 0.676) or BA preference (p = 0.076) were significantly changed. However, the naïve salt preference was significantly different from CantonS control larvae (p = 4.9×10^−5^). As CantonS larvae showed the lowest salt avoidance of all measured genotypes, the significant difference may, at least partially, be due to the low performance of the control larvae. Therefore, we also suggested that apart from the distinct appetitive learning phenotype, aversive memory impairment of the *dumb^1^* mutant was also attributable to an impairment of acquisition and/or retrieval, rather than to changes in sensory perception. For *DAMB*, neither salt avoidance (p = 0.651) nor BA preference (p = 0.858) were significantly different compared to *rosy* control larvae. However, sugar perception (p = 0.002) and AM preference (p = 0.005) were significantly reduced compared to *rosy* controls. Nevertheless, *DAMB* larvae were still able to perceive sugar (p = 0.017) and AM (p = 0.003) and were able to form a normal appetitive olfactory memory (Figure 4D). Therefore we suggest that the aversive memory impairment of *DAMB* was attributable to an impairment of the acquisition and/or retrieval, rather then to changes in sensory perception (although this cannot fully be excluded).

Taken together, our behavioral approaches based on the presynaptic block of DA signaling and DA receptor mutants may suggest that DA is not only involved in aversive but also in appetitive olfactory learning. However, when blocking DA neurons, changes in the sensory acuity of the animals may, at least partially, interfere with these results. Differential effects, when comparing DA receptor mutants and blocking synaptic output of TH-GAL positive neurons with respect to larval learning may have several underlying reasons: (i) other DA receptors exist apart from the two analyzed receptors; (ii) TH-GAL4 did not exclusively label DA neurons; (iii) TH-GAL4 did not cover all DA neurons. To distinguish between these possibilities, cellular dissection of the DA system is required.

### Anatomy of the Dopaminergic System in the Larval CNS at the Single Cell Level

What could be the anatomical substrates that mediate aversive and appetitive learning in the DA system? As its architecture was complex (Figure 2) we labeled individual TH-GAL4 positive neurons by using the flp-out technique [52]. This allowed us to identify the morphology of single neurons by anti-GFP staining in the background of anti-FasII/anti-ChAT axonal tracks/neuropile staining. We focused on neurons that, based on their anatomy, could be potential candidates for processing gustatory information from the sog onto the olfactory pathway. Given the limited innervation of the al by DA neurons, if at all, we concentrated on the mb Kenyon cells, the third order-neurons of the olfactory pathway. This strategy was further supported by the strong dDA1 and DAMB antibody staining in the mbs (Figure 4F, 4H and 4J) suggesting output of the DA system onto cells in this neuropile. We generated more than 400 single, double or multi-cell clones. Table 2 comprises the cell types and their innervation pattern in the central brain regions, mbs, sog and vnc. It also shows the number of hits per cell type and the double flp-out cases, which provide evidence about the paired nature of a given type of neuron. In total we collected 274 brains that allowed us to follow the projections of single neurons.

**Table 2:** Innervation patterns of the TH-GAL4 cell types, described by single cell staining.

cell type	dmp	dlp	bmp	blp	ma	vl	la	sp	ped	ca	sog	tg	ag	hits per cell	paired in one brain
														n = 274	n = 274
**DL1-1**	***il***					***sm***								14	
**DL1-2**	***il***					***sm***								9	
**DL1-3**	***il***		***bs***			***cl***								14	X
**DL1-4**	***il***		***il***				***sm***							29	
**DL1-5**	***il***							***sm***	***sm***					12	
**DL1-6**	***il***		***sm***											11	
**DL2-1**	***bs***								***bs***	***bs***				13	X
**DL2-2**	***il***	***il***								***il***				25	X
**DL2-3**			***bs***	***bs***							***il***			32	X
**DM1**			***il***	***il***				***il***			***il***	***il***		13	X
**DM2**	***il***				***sm***									1	
**DM3**	***bs***	***il***								***il***				16	X
**DM4**	***il***	***il***												1	
**DM5**				***il***							***il***			20	
**SM1-1**			***sm***								***sm***	***sm***		8	
**SM1-2**											***bs***			36	XX
**SM2-1**			***sm***								***sm***			14	
**SM2-2**			***il***								***il***	***il***	***il***	15	X
**SL1**											***bs***			16	X
**SL2**											***bs***			33	X
**TM1-1**											***sm***	***sm***		3	
**TM1-2**			***il***								***il***	***il***	***il***	13	X

central brain regions: dmp- dorsomedial, dlp- dorsolateral, bmp- basomedial, blp- basolateral protocerebrum; mushroom bodies: ma- medial appendix, la- lateral appendix, vl- vertical lobe, sp- spur, ped- pedunculus, ca- calyx; sog- suboesophageal ganglion; ventral nerve cord: tg- thoracic ganglion, ag- abdominal ganglion; DL- dorsolateral, DM- dorsomedial, SM- SOG medial, SL- SOG lateral, TM- thoracic medial; symmetrical (sm), unsymmetrical: both sides (bs), ipsilateral (il), contralateral (cl).

**Figure 5:**
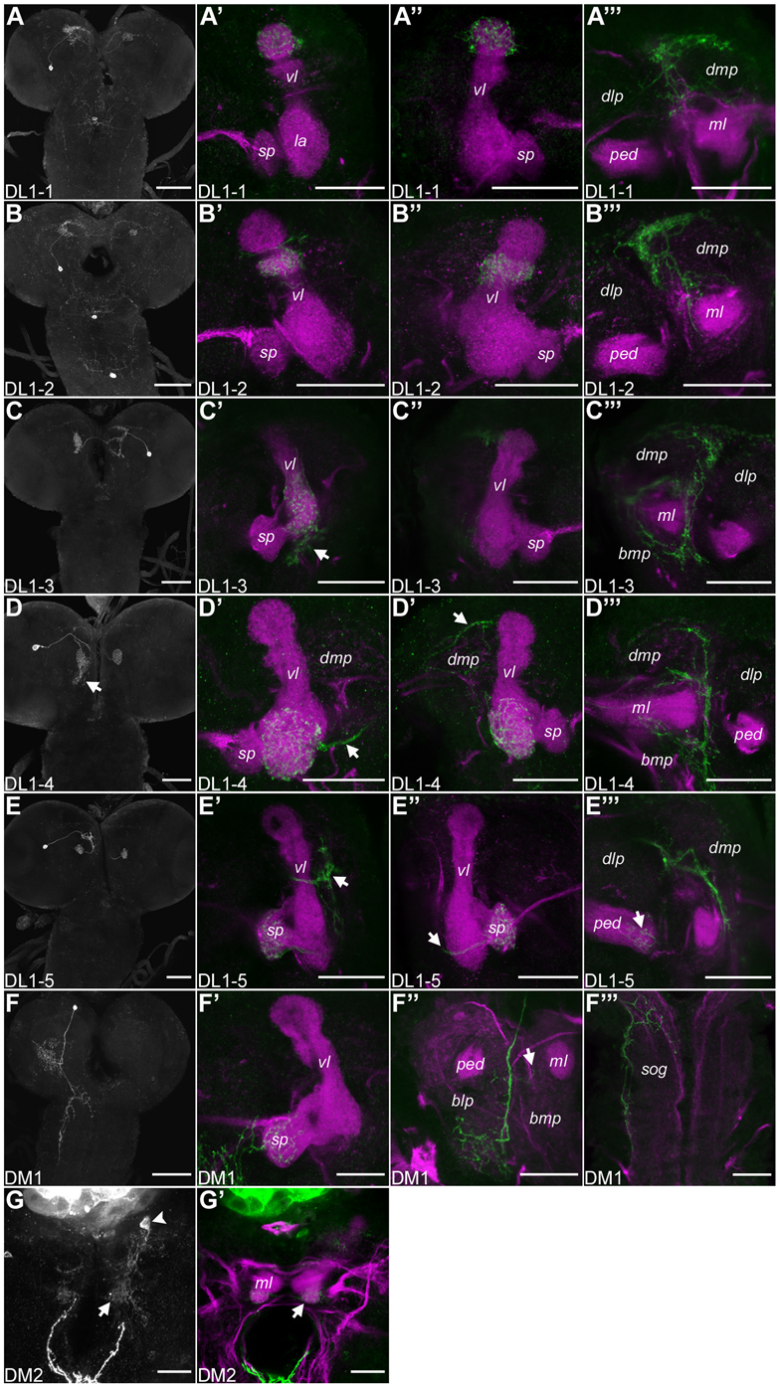
Single Cell Staining of Potentially Dopaminergic Neurons Innervating the Mushroom Body. The left column shows a projection of the hemispheres and the sog of the anti-GFP labeling for each cell type. The other columns represent higher magnifications of specific neuropile regions (magenta) innervated by the respective cell type of the TH-GAL4 line (green). (A–A″) The DL1-1 neuron innervates the most dorsal parts of the vertical lobes (vls). (A′″) A branch of the primary neurite bifurcating posterior to the ipsilateral vl ramifies in the dorsomedial protocerebrum (dmp). (B–B″) DL1-2 innervates the vls ventral to the most dorsal tip. (B′″) Ramifications of DL1-2 in the ipsilateral dmp. (C–C′″) DL1-3 innervates the contralateral vl. The neurite crossing the midline bifurcates in the dorsal part of the vl posterior to the lateral appendix (la) and the basal protocerebrum (arrow C′). (C′″) Innervation of the ipsilateral dmp and basomedial protocerebrum (bmp) posterior to the vl. (D–D′″) DL1-4 (the higher magnifications in D′–D′″ are from a different brain than the total projection pattern in D) projects to the la (D″), the dmp (arrow D″; D′″) and the bmp (arrow D; D′″). An axon crosses the midline (arrow D′) and terminates in the contralateral la. (E–E′″) DL1-5 ramifies in the ipsilateral dmp (arrow E′) and the spur (sp). (E″) An axon terminates in the contralateral sp (arrow). (E′″) The anterior pedunculus (ped) shows arborizations (arrow). (F–F′″) DM1 shows only ipsilateral innervation in the central brain, sog and thoracic ganglion. (F′) The sp of the mb is innervated. (F″) The primary neurite ramifies in the posterior basolateral protocerebrum (blp). Filiform branches are sent in the posterior bmp (arrow). (G,G′) DM2 innervates the medial appendices (ma; arrows). Scale bars: left column 50 µm all other 25 µm.

**Figure 6 pone-0005897-g006:**
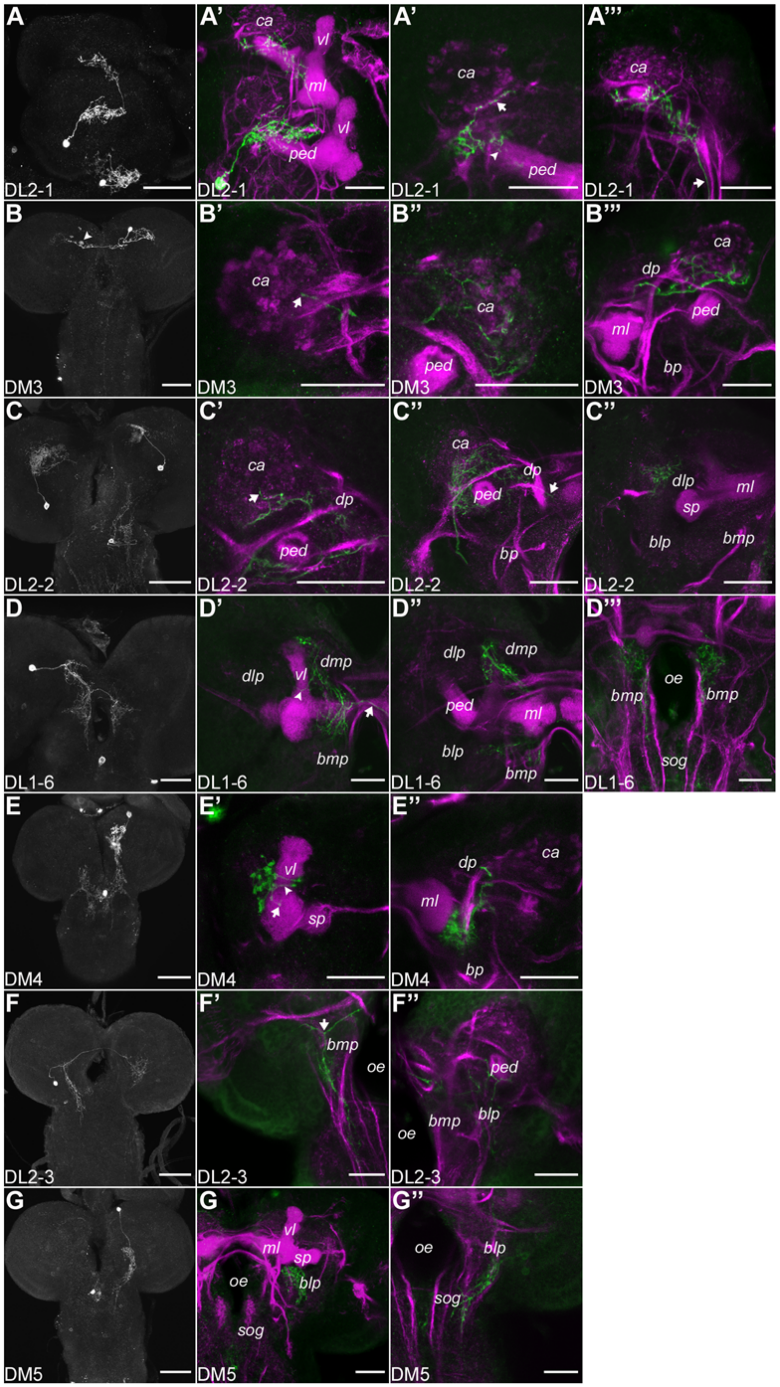
Single Cell Staining of Potentially Dopaminergic Neurons. The left column shows the projection pattern of each cell type. The other columns represent higher magnifications of neuropile regions (magenta; A″, B″, C′ and G″ show single confocal sections) innervated by the respective cell type (green). (A–A′″) Sagittal view of the innervation pattern of the DL2-1 cell type. DL2-1 projects to both calyces (ca; arrow A″ and A′″) and ramifies in the dorsal protocerebrum (dp). The posterior pedunculi (ped) show innervations (arrowhead A″). (B–B′″) Two neurons of the DM3 cell type one of which is stained much weaker (arrowhead). (B′) The axon crossing the midline terminates at the contralateral ca (arrow). The ipsilateral ca and dp are innervated (B″ and B′″). (C–C″) The ipsilateral projecting DL2-2 cell type innervates the basolateral/mediolateral ca (arrow C′). Ramifications in the dorsolateral protocerebrum (dlp) were observed (C′–C′″). DL2-2 bifurcates around the ped (C′ and C″) and reaches the neuropile lateral to the posterior part of the medial lobe (ml; arrow C″). (D–D′″) DL1-6 shows the characteristic dorsomedial protocerebrum (dmp) innervation. A small axon projects through the vertical lobe (vl) without any ramifications (arrowhead D′). The basomedial protocerebra (bmp) are innervated. (E–E″) DM4 innervates the dp around the vl. (E′) Two axons project through the lobe (arrowhead). A dot-like terminal is observed in the lateral appendix (arrow). (E″) The protocerebrum lateral to the ml is innervated. (F–F″) The innervation of DL2-3 is restricted to the basal protocerebrum (bp). The primary neurite bifurcates in the posterior bmp (arrow F′). One secondary neurite innervates the bp and posterior sog (F′). The other is sent to the contralateral side and ramifies in the bp (F″). (G–G″) DM5 innervates the anterior basolateral protocerebrum (blp) ventral to the vl. (G″) Single section of the posterior blp and posterior sog. Scale bars: left column 50 µm, rest 25 µm.

**Figure 7:**
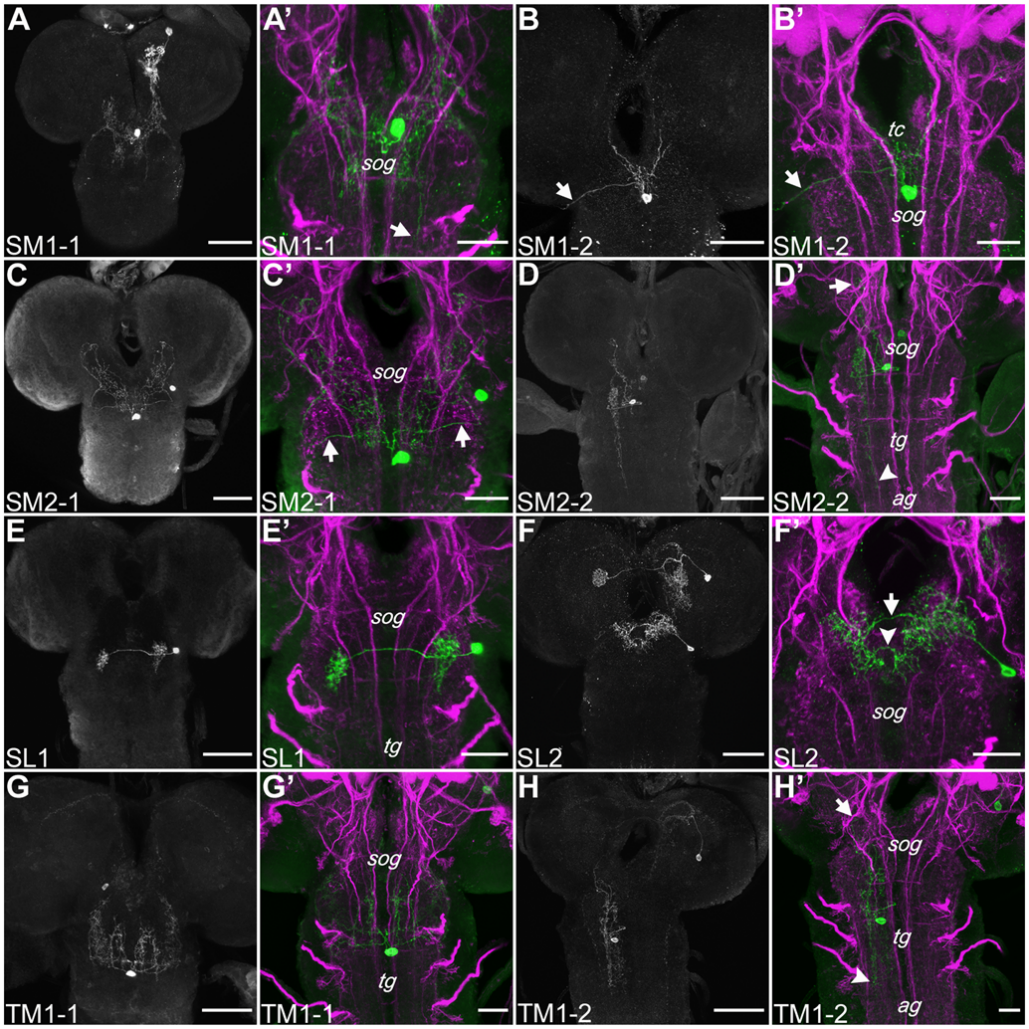
Single Cell Staining of TH-GAL4 Neurons of the Suboesophageal and Thoracic Ganglion (S and T Clusters). The grayscale pictures show the projection pattern of each cell type. The other pictures show higher magnifications of neuropile regions (magenta) innervated by the respective cell type (green). (A–F′) Cell types of the sog clusters. (A and A′) SM1-1 innervates the sog, the basomedial protocerebra (bmp) posterior to the antennal lobes and shows terminals in the anterior thoracic ganglion (tg; arrow A′). (B and B′) This paired hugin cells innervate the tritocerebrum (tc) and send an axon to the periphery (arrows B and B′). (C and C′) The unpaired neuron innervates the sog and lateral and ventral parts of the bmp. Two secondary neurites run at the posterior margin of the sog laterally (arrows C′). (D and D′) The paired SM2-2 shows a dense innervation in the lateromedial sog which goes further basal to the thoracic and abdominal ganglia (ag; arrowhead D′). The neuron projects to the bmp (arrow D′). (E and E′) SL1 innervates the medial part of the basolateral sog on both sides of the cns. The arborizations are connected through a single axon crossing the midline. (F and F′) SL2 sends fibers in the ipsilateral dorsoanterior sog, projects over the midline (arrow F′) and bifurcates in the contralateral anteriomedial sog. Another axon crosses the midline basal to the primary neurite (arrowhead F′) and terminates at the ipsilateral side. (G–H′) Neurons of the medial cluster in the first thoracic segment (TM1). (G and G′) The cell body of the unpaired TM1-1 neuron is located at the ventromedial tg. The anterior part of the first thoracic segment and basal sog are innervated. (H and H′) TM1-2, a paired cell, sends arborizations in the medial and lateromedial sog, ventromedial tg and anterio-ventromedial ag (arrowhead H′). Branches in the bmp are observed (arrow H′). Scale bars: overview 50 µm, higher magnification 25 µm.

The maximally eight stained neurons of the DL1 cluster could be categorized into six different types (DL1-1–DL1-6). As the DL1-4 type was hit twice as often as the others (Table 2), we speculated that it was represented twice in the DL1 cluster. The DL2 cluster, which consisted of about six neurons, seemed to be organized in three different cell types. Interestingly, two types occurred more often in our flp-out clones; therefore we speculated that these were also represented twice per hemisphere (DL2-2 and DL2-3; Table 2). Yet, we cannot exclude that we missed one cell type in the DL2 cluster. For the DM cluster, we were able to identify five different types of neurons. However, due to the weak expression in other cells, we were not able to classify all of these neurons; here the flp-out technique displayed obvious limitations. In the SM clusters we identified all TH-GAL4 positive neurons, which belonged to four different types. Multi-cell clones suggested that one type occurred four times (SM1-2; Table 2). For the SL cluster we described two types. Again one type emerged more often in our analysis, suggesting that it comprises at least two copies (SL2; Table 2). In total, we identified a comprehensive set of TH-GAL4 positive neurons, leaving only weakly labelled neurons unidentified due to the limitations of the flp-out technique (Figures 5, 6 and 7). At least 19 different types of TH-GAL4 positive, mostly paired neurons were found to innervate the brain, for example the mbs; twelve types innervated the sog (Figures 5, 6 and 7). Remarkably, none of these neurons innervated both the mb and GRN input region of the sog. Next, we focused on candidates potentially involved in aversive and appetitive olfactory learning, i.e. neurons having their cell body in the hemispheres, sog or thoracic ganglion. The terminology used refers to their association with the DL, DM, S or T clusters, followed by a further subdivision into different types. The following sections first describes cells innervating the mb lobes and/or the pedunculi (Figure 5), then those sending their branches onto the calyces (Figure 6A–6C), and finally the neurons that may have a limited mb projection (Figure 6D–6G).

DL1 neurons (Figure 5) were characterized by a dorsally projecting primary neurite passing laterally around the calyx and bifurcating posterior to the vertical lobe. The terminal branches innervated the dorsomedial protocerebrum and a region of the lobes specific for each DL1 subtype. One axon crossed the midline parallel to the dorsoposterior commissure, and terminated at the contralateral lobe in a mirror-symmetric pattern compared to its ipsilateral lobe innervation. The **DL1-1** neuron (Figure 5A–5A′″) projected onto the tips of both vertical lobes (Figure 5A′ and 5A″). The primary neurite bifurcated posterior to the vertical lobe; arborizations were observed in the ipsilateral dorsomedial protocerebrum (Figure 5A′″) and the dorsalmost part of the vertical lobe (Figure 5A′). An axon crossed the midline along the dorsoposterior commissure and branched at the dorsal tip of the contralateral vertical lobe (Figure 5A″). The ipsilateral dorsomedial protocerebrum was also innervated by **DL1-2** (Figure 5B–5B′″). A secondary neurite crossed the midline and terminated beneath the tip of the vertical lobe (Figure 5B″). Ipsilateral and contralateral innervations of the vertical lobes were overlapping (Figure 5B′ and 5B″). **DL1-3** (Figure 5C–5C′″) was somewhat atypical for DL1 cells because it mainly projected to the contralateral mb (though small terminals at the posterior margin of the ipsilateral lobe were not excluded). The ipsilateral innervation was restricted to the dorsomedial and parts of the basomedial protocerebrum, posterior and lateral to the mb (Figure 5C′″). Contralaterally, DL1-3 ramified in the ventral part of the vertical lobe, posterior to the lateral appendix and showed no overlap with the terminals of DL1-4 (data not shown). A small region of the protocerebrum basal to the lobes was also innervated (arrow Figure 5C′). **DL1-4** (Figure 5D–5D′″) projected to the lateral appendices of the mbs. The primary neurite extended dorsally, bifurcated in the lateral appendix and the basomedial protocerebrum (arrow Figure 5D), while a secondary neurite emerged posterior to the vertical lobe and crossed the midline (arrow Figure 5D′). The dorsomedial protocerebrum showed the characteristic innervation of the DL1 cell types (Figure 5D″). **DL1-5** had arborizations in the ipsi- and contralateral spurs of the mbs and the anterior parts of the pedunculi (Figure 5E–5E′″) and in the ipsilateral dorsomedial protocerebrum (arrow Figure 5E′). This cell type showed a particular innervation pattern in the dorsomedial protocerebrum, as it bifurcated lateral to the vertical lobe and sent an axon through (and perhaps even synapsing with) the vertical lobe.

Two neurons of the DM cluster also innervated parts of the lobes and/or pedunculi. Arborizations of **DM1** (Figure 5F–5F′″) were restricted to the ipsilateral side of the brain, sog and thoracic ganglion. The primary neurite extended ventrally and bifurcated in the basolateral protocerebrum, whereas the basomedial protocerebrum was innervated by only one small axon (arrow Figure 5F″). The mb spur was densely innervated and a single fiber was sent into the lateral appendix (Figure 5F′). An axon projected further ventral and branched in the dorsal thoracic ganglion (Figure 5F′″). DM1 arborizations in the posterior sog were unlikely to overlap with GRN terminals as they were shown to be located more anteriorly in the sog [14]. The **DM2** cell (Figure 5G and 5G′), which was found only once, was weakly stained. Yet, we think that it projected to both medial appendices - by crossing the midline ventral to the medial lobe - and innervated at least the ipsilateral dorsomedial protocerebrum.

The next section describes TH-GAL4 neurons that potentially arborized in the mb calyx (similar to the innervation described in adults [56]). They seemed to have their cell bodies in the DL2 and DM clusters (Figure 6A–C) and to restrict their terminals to the lateral part of the calyx. **DL2-1** (Figure 6A–6A′″; pictures show a brain from a lateral view) terminated on the lateral calyces and the posterior parts of the pedunculi. It arborized in the dorsomedial protocerebra on both sides of the brain. Ipsilateral branches also covered the protocerebrum around the posterior part of the pedunculus. **DM3** (Figure 6B–6B′″) was characterized by ramifications of a laterally projecting axon which densely innervated the most anterior part of the ipsilateral calyx (Figure 6B″) and also reached the dorsomedial and dorsolateral protocerebra anterior to the calyx (Figure 6B′″). A secondary neurite projected across the midline and it terminated in the lateral part of the contralateral calyx (arrow Figure 6B′). In addition, small branches in the dorsoposterior protocerebrum were observed. **DL2-2** (Figure 6C–6C″) remained strictly ipsilateral. Its primary neurite projected dorsally, bifurcated and terminated widely in the dorsolateral protocerebrum including the lateral horn, around the pedunculus and in the anteriolateral calyx (arrow Figure 6C′ and 6C″). The dorsomedial protocerebrum was innervated by small arborizations, mainly lateral to the pedunculus. DL2-2 also reached the neuropile lateral to the posterior part of the medial lobe (arrow Figure 6C″).

For the following cell types, terminals in the mbs were less obvious, as their overlap with the neuropile markers in this region, was very limited. This overlap mainly consisted of small side branches. The primary neurite of **DL1-6** (Figure 6D–6D′″) bifurcated posterior to the ipsilateral vertical lobe and terminated in the dorsomedial protocerebrum and around the vertical lobe (Figure 6D′ and 6D″). From a dense arborization anterior to the medial lobe small branches extended to the lateral part of the lobe (Figure 6D′). An axon crossed the midline (arrow Figure 6D′) and innervated the basomedial protocerebrum, mainly around the oesophagus. From a somewhat similar ipsilateral arborization small axons projected to the posterior sog (Figure 6D′″). **DM4** (Figure 6E–6E″) was hit only once but simultaneous visualization of a neuron in the sog did not allow us to comment on the innervation in the basal protocerebrum. Neurites ramified lateral to the medial lobe of the mb, projecting also ventral (Figure 6E″). Around the vertical lobe terminals built a dot-like structure mostly in the dorsal protocerebrum (Figure 6E′). The arrow in Figure 6E′ showed potential presynapses on the ventral part of the vertical lobe. The primary neurite of **DL2-3** (Figure 6F–6F″) turned in the basolateral protocerebrum toward the midline, bifurcated before reaching the oesophagus (arrow Figure 6F′), then innervated the basal protocerebrum and finally reached the posterior sog. A secondary neurite extended contralaterally via the dorsoposterior commissure. The dorsal parts of the basolateral protocerebrum were innervated and the contralateral pedunculus was surrounded by terminals (Figure 6F″). **DM5**, an ipsilaterally-projecting neuron of the DM cluster (Figure 6G–6G″) sent its primary neurite to the basolateral protocerebrum (Figure 6G″). Branches projected posterior and innervated the dorsoposterior sog. Other branches ramified ventral to the spur and the lateral appendix (Figure 6G′).

In addition we found eight cell types innervating the sog (Figure 7); none of which projected to the mb. However, four TH-GAL4 positive neurons overlapped with neurons expressing the hugin neuropeptide, members of a neural circuit that modulates taste-mediated feeding behavior (identified by double labeling [89]; loc. cit. Figure 4D and 4E). The SM1 cluster contained four to five neurons whereas the SM2 and TM1 clusters included three cells each (Figure 7A–7 D′ and 7G–7H′).

The cell body of the **SM1-1** neuron was located at the midline in the most anterior part of the sog (Figure 7A and 7A′). Its primary neurite ran posteriorly along the midline, split and sent two axons laterally, which bifurcated in the central part of the sog. Two axons turned laterally in the most posterior sog; they innervated the lateral sog margin. SM1-1 also arborized in the basomedial protocerebrum posterior to the al and sent small fibers to the thoracic ganglion (arrow Figure 7A′). Based on double labeling experiments, the paired **SM1-2** neurons (Figure 7B and 7B′) were already described previously [89], [90] as hugin cells. In our preparations, they innervated the tritocerebrum and sent an axon to the pharynx (arrows Figure 7B and 7B′). We noted that these cells, although labeled by TH-GAL4, were not TH-positive (arrow Figure 2G; the two fiber like structures labeled in the dorsalmost part of the brain presumably belonged to SM1-2 neurons projecting to the pharynx). The primary process of **SM2-1** whose cell body was situated at the midline in the anterior basal sog (Figure 7C and 7C′) extended posterior, split in two neurites, which turned laterally in the posterior sog (arrows Figure 7C′). At its lateral margin they twirled dorsal to the intersection between the sog and the basal protocerebra. The neuron innervated the posteriomedial sog and the lateral and ventral parts of the basomedial protocerebra. The paired **SM2-2** neurons (Figure 7D and 7D′) also sent a process to the posterior margin of the sog. But different to the unpaired cell types, the neurite ran laterally to the midline. SM2-2 showed a dense innervation in the basomedial protocerebrum (arrow Figure 7D′) and the lateromedial sog which further extended to the thoracic and abdominal ganglia (arrowhead Figure 7D′). The innervation pattern of the paired cells of the SL cluster seemed to be restricted to the sog (Figure 7E–7F′). **SL1** showed bilateral arborizations in the medial region of the basolateral sog (Figure 7E and 7E′). The ipsilateral innervation reached the most posterior part of the lateromedial sog, whereas the contralateral bifurcations were restricted to the anterior sog. **SL2** was the only type of TH-GAL4 neurons that innervated the dorsoanterior sog (Figure 7F and 7F′). Its primary neurite sent fibers in the ipsilateral dorsoanterior sog, crossed the midline (arrow Figure 7F′) and bifurcated in the contralateral anteriomedial sog. Another axon crossed the midline basal to the primary neurite (arrowhead Figure 7F′) and terminated at the ipsilateral side. The cell body of the unpaired **TM1-1** neuron was located in the ventromedial thoracic ganglion. Its primary process split into four secondary neurites, two of them ran along the midline further dorsal whereas the others projected laterally (Figure 7G and 7G′; see [91]). Secondary neurites innervated the anterior part of the first thoracic segment and the basal sog. The paired **TM1-2** neurons, whose cell bodies were located at the ventromedial side of the first thoracic segment [see also 85] projected dorsally next to the midline and, upon reaching the dorsal margin, extended laterally sending arbors in the medial and lateromedial sog, the ventromedial thoracic and anterior-ventromedial abdominal ganglion (arrowhead Figure 7H′), as well as in the basomedial protocerebrum (arrow Figure 7H′).

## Discussion

### The Role of the Dopaminergic System in Adult and Larval *Drosophila*

DA, which is present in relatively high concentrations in the *Drosophila* brain, was suggested to play an important role as a neurotransmitter and/or neuromodulator [36], [38], [63], [83], [92]. During *Drosophila* development, DA levels show discrete peaks. These coincide with larval moults, pupariation and adult emergence consistent with the finding that DA is required in insects for cuticle hardening and pigmentation [93]. The analysis of *Drosophila* mutants also suggests a role for DA in the terminal differentiation of the nervous system [38]. Apart from these developmental aspects, the DA system of *Drosophila* is involved in many acute behavioral functions such as ethanol-induced courtship disinhibition [94], experience-dependent plasticity in sleep [95], arousal [96], [97], akinesia, developmental retardation, decreased fertility [98], locomotion, stereotyped behaviors like grooming [99], [100] and saliency-based decision-making [101]. For the present study, the requirement of DA in learning and/or memory retrieval [30], [34], [35] is especially important.

**Figure 8:**
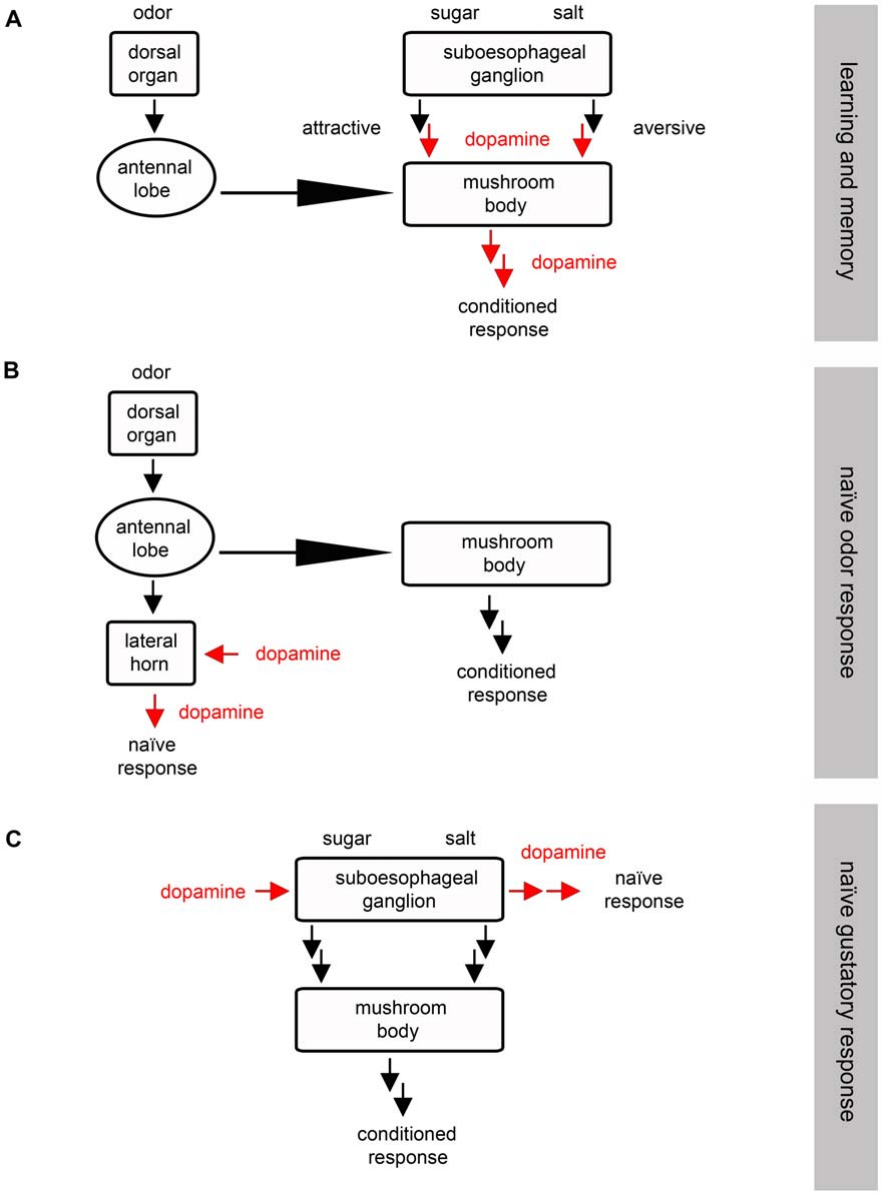
DA is Involved in Olfactory Learning, Naïve Odor and Naïve Taste Responses. Olfactory stimuli are detected by the dorsal organ and transmitted via the antennal lobe to the mb and lateral horn. The mb is postulated as a site of coincidence detection of odor signals from the antennal lobe and salt or sugar signals from the sog. The lateral horn and the sog might be involved in mediating naïve odor responses and naïve gustatory preferences, respectively (A–C). (A) We postulate a role of DA in aversive and appetitive olfactory learning, interfering either with aversive and appetitive reinforcement signaling, or with memory retrieval. DA neurons innervating the lateral horn (B) may be involved in naïve odor responses whereas DA neurons innervating the sog (C) may control naïve gustatory responses.

We found that blocking output from TH-GAL4 positive neurons impairs associative aversive scores (Figure 3H), but leaves responsiveness to the to-be-associated stimuli (1-octanol: Figure 3L; salt: Figure 3N) intact. The same defect in associative aversive scores is seen in the DA receptor mutants *dumb^1^*, *dumb^2^* and *DAMB* (Figure 4A and 4C). Thus, together with the previous reports of Tempel et al. [102], Schwaerzel et al. [30], Kim et al. [35] and Honjo and Furukubo-Tokunaga [33], the requirement of dopaminergic signaling for associative aversive conditioning seems well substantiated (Figure 8A).

Regarding the cellular identity of the required dopaminergic signal, the TH-GAL4 strain obviously covers a set of required neurons (this study; [30]); the same strain also covers at least one neuron, which impinges onto the mbs and is activated by an electric shock stimulus [103]. Thus, because Kim et al. [35] found that in dDA1 mutant flies the impairment in the aversive paradigm can be rescued by receptor expression in a subset of mb Kenyon cells, it seems plausible that DA input onto the mbs serves as an obligatory reinforcement signal for the acquisition [30] of aversive olfactory memory traces. This scenario seems to apply to insects in general, as argued from pharmacological studies in honeybees and crickets [31], [32]. For example, in honeybee sting extension reflex conditioning it was shown that injection of the DA receptor antagonist flupentixol, but not of the OA receptor antagonist mianserine nor of ringer solution, impaired bees of learning to discriminate a reinforced from a non-reinforced odorant [32]. Crickets injected with the OA receptor antagonists epinastine or mianserine showed impaired appetitive learning with water reward, while aversive learning with saline punishment remained intact. In contrast, fluphenazine, chlorpromazine or spiperone, all DA receptor antagonists, impaired aversive learning without affecting appetitive learning [31]. Much in contrast, it seems that in mammals DA is acting as reinforcement signal during appetitive, but not during aversive learning [104]. It was therefore an attractive thought that between mammals and insects the role of dopamine as reinforcing signal is conserved, and that ‘only’ the valence of the signal is reversed. Indeed, in insects another biogenic amine, OA, is required for appetitive learning [bee: 28], [29], [105], [106; cricket: 31], [Bibr pone.0005897-Unoki1] [107]; [*Drosophila*: 30]; in crickets the necessity of OA for mediating positive reinforcing signals can even be generalized to learning of sensory signals other than odors. Crickets injected with epinastine or mianserine, OA receptor antagonists, exhibited a complete impairment of appetitive learning, i.e., associating a visual pattern with water reward, while DA receptor antagonists fluphenazine, chlorpromazine or spiperone did not affect appetitive learning [107]. Actually, OA may indeed be sufficient as an appetitive reinforcement signal in the bee [28]; [regarding Drosophila: 34]. Given that in turn the animals appear unimpaired in the appetitive paradigm upon distortion of OA signaling, one wonders whether indeed the role of DA is selective for aversive paradigms or not.

### Requirement of Dopamine-Signaling for *Drosophila* Appetitive Learning, too?

We found regarding larval *Drosophila* that blocking output from TH-GAL4 neurons impairs the animals in the aversive and potentially also in the appetitive paradigm (Figure 3I); however, under these conditions the response towards the sugar reward is also impaired (Figure 3M), which makes this defect difficult to interpret. On the other hand, this suggests that DA neurons may be involved either in processing of odor and sugar stimuli or alternatively in mediating appropriate naïve responses to odors and sugar. However, the DA receptor mutants *dumb^1^* and *dumb^2^* also show defects in the appetitive paradigm (Figure 4B), without concomitant defects in terms of their responses to the to-be-associated stimuli (Figure 4K–4N). Together with the observation that adult *dDA1* mutants are impaired in the appetitive paradigm, and that this defect can be rescued by expressing the receptor in a subset of mb Kenyon cells [35], this suggests that DA signaling is required for establishing and/or retrieving an appetitive olfactory memory trace, and that likely DA input onto the mbs is critical in this regard. Such scenario apparently is at variance with the reports of Schwaerzel et al. [30] in adult flies and Honjo and Furukubo-Tokunaga [33] in larvae, both of which did not observe any defect in appetitive conditioning when DA neurotransmission was blocked in TH-GAL4 positive neurons [30], [33]. This discrepancy could be due to (i) differences in the expression level of TH-GAL4 because of different insertion sites of the transgene; (ii) more efficient block in our experiments due to the pre-incubation at 37°C and a higher restrictive temperature (34°C), compared to no pre-incubation and lower restrictive temperature (31°C) in the previous studies [30], [33]. Interestingly, Rister et al. [108] used 15–20 min pre-incubation at 37°C to fully block different types of laminar neurons in the optic lobes using UAS-*shi^ts1^*. Moreover, Song et al. [109] reported that a 20 min latency at 37°C is required to fully immobilize larvae when expressing UAS-*shi^ts1^* in all peripheral sensory neurons; this is similar to our findings after blocking olfactory receptor neurons. Upon comparing these observations with the original studies by Kitamoto [50], [51] it is tempting to speculate that the efficiency of shibire^ts1^ neurotransmission block depends on the type of neuron. And only regarding the report of Schwaerzel et al. [30] differences compared to our data might depend on (iii) DA neurons included in the expression pattern of TH-GAL4 that are specifically involved in larval, but not in adult appetitive olfactory learning; (iv) those neurons that are critical during the larval stage may not be part of the expression pattern anymore in adults. While any of these explanations may be true, none of them would explain the requirement of dopamine receptors for the appetitive paradigm. Given the fair specificity of these receptors for dopamine [40], [43] one may alternatively contemplate whether the relatively low learning scores for appetitive learning as reported in Schwaerzel et al. ([30] corresponding to 0.15 in the UAS-*shi^ts^* control; loc. cit. Figure 4), together with a relatively low sample size of merely six may not have unwittingly overlooked a defect in the appetitive paradigm. Clearly, the available data do not allow pitting these various accounts against each other.

### Sufficiency of Dopamine-Signaling for *Drosophila* Aversive and Appetitive Learning?

Notably, *Drosophila* larvae establish an aversive olfactory memory when TH-GAL4 neurons are experimentally activated together with an odor stimulus [34]. These experiments, much like ours, refer to the whole complement of DA neurons covered by TH-GAL4; thus, a subset of these neurons may be involved in aversive learning, whereas another subset may be involved in appetitive learning. The net effect would be aversive when all these neurons are activated (Figure 8A). Alternatively, TH-GAL4 expression may include some neurons that are sufficient for aversive reinforcement during training; in addition these neurons may serve the same purpose with regard to appetitive learning. Indeed, Schroll et al. ([34]; loc. cit. Figure S1) showed that larvae, in which TH-GAL4 neurons are experimentally activated together with an odor stimulus, tend to display an appetitive memory when tested in the absence of salt. Clearly, tackling these kinds of questions calls for a detailed understanding of the anatomy of the TH-GAL4-positive neurons on the single-cell level.

### Dopaminergic Circuitry of the Larva

The DA neurons of the larval brain belong to distinct clusters in the brain hemispheres, sog and thoracic ganglia (Figure 5–7), which we describe here in detail on the single-cell level. A detailed overview of the different cell types and their innervation of different areas in the central brain, sog and vnc is given in Table 2. By and large, neurons from the DL1 cluster preferentially innervate the mbs either in the vertical lobes, the lateral appendices, spurs and pedunculi, whereas DL2 neurons project to the mb calyx and pedunculus. Regarding the cells in the DM cluster, the morphology of the different types of neurons is too diverse to allow similarly generalized statements. Thus, the larval DA system is obviously connected to the mbs, apart from a remarkably defined innervation of the sog and the protocerebrum. Indeed, in the larva both DA receptors dDA1 and DAMB are highly expressed in the mbs; also, the strong expression of presynaptic markers in DA neurons in the vertical lobes, medial lobes, medial and lateral appendices, spurs and pedunculi indicate DA neuron output at the mb level. Finally, the morphology of single neurons suggests a bouton-like structure of certain dopaminergic terminals in the mbs (Figure 5 and 6). Notably, none of the candidate mb-innervating DA neurons seems to receive input directly in the sog. Thus, DA neurons are obviously not exclusive gustatory projection neurons, but may be integrating signals across a broader range of inputs onto the mbs.

Given the possibility that some TH-GAL4 neurons may act during memory retrieval (Figure 8A), we note that some of the mb innervating DA neurons have overlapping expression in the dorsal protocerebrum. Furthermore, DA neurons also innervate defined areas in the basal protocerebrum (Figure 5 and 6). Therefore it is tempting to speculate that the protocerebrum may contribute to express a behavior but not to establish a memory. Compatible with this idea it was shown in larval olfactory learning the relevant behavior (e.g. movement towards an odor) is not simply a passive, stimulus-evoked process but is expressed only, if the outcome offers a benefit for the larvae [1]. Also, in honeybees reserpine depletes biogenic amines from their stores in the brain and leads to impaired appetitive conditioning. Compensatory injection of DA directly into the brain rescues the slowdown effect on motor patterns, but not sensitization or conditioning [105]; [see also 110], [111] [112].

### Outlook

Together, it seems clear that DA signaling has more than one function hitherto ascribed to it, namely to convey an aversive reinforcement signal during punishment learning. Rather, we suggest that DA signaling plays a similar role in appetitive paradigms, and conceivably in the retrieval of both aversive and appetitive memory, apart from its potential role in sugar and odor perception (Figure 8B and 8C). Given our anatomical evidence that DA signals may reach the critical ‘olfactory memory center’ (the mb) via multiple routes, we propose that different types of DA neurons might be involved in different types of signaling necessary for aversive and appetitive olfactory memory formation, respectively, or for the retrieval of these memory traces.

We believe that future studies of the DA system need to take into account such cellular dissociations in function in order to be meaningful.

## Materials and Methods

### Fly Strains

Fly strains were reared on standard *Drosophila* medium at 25°C or 18°C with a 14/10 h light/dark cycle or in constant darkness in case of the hsp70-flp ; TH-GAL/+ ; UAS>CD2y^+^>mCD8::GFP/+ flies. For the behavioral experiments, UAS-*shi* ^ts1^ [50], with an insertion on the third chromosome was used as an effector to block defined neurons by crossing to the GAL4-driver line TH-GAL4 [36]. Heterozygous controls were obtained by crossing GAL4-driver and UAS-effector to *w^1118^*. We also used mutant strains *DAMB*, *dumb^1^*, *dumb^2^* [41] and control lines CantonS, *w^1118^* and r*osy*. For visualizing neurons, we crossed TH-GAL4 with UAS-*Cameleon2.1* [64] and UAS-*mCD8::GFP* [52]. The pre- and postsynaptic regions of the TH-GAL4 expressing neurons were labeled using UAS-*nsyb::GFP* [74], [75] or UAS-*Dscam[17.1]::GFP* [72]. For the single cell staining *y w hsp70-flp; Sp/CyO*; *UAS>CD2y^+^>mCD8::GFP/TM6b* [52] virgins were crossed to TH-GAL4 males. A single heat shock at 37°C for 18 min was applied by placing the vials in a water bath. For the onset of heat shock, we chose different times from 0 to 200 hours after egg laying.

### Behavioral Experiments

For the learning assays, flies were allowed to lay eggs for two days. Experiments were performed at the fifth or sixth day after beginning of egg laying. Third instar larvae used for the behavioral experiments were therefore 96–144 hours old; only feeding stage larvae were taken. For preparing the assays, 2.5% agarose solution (Sigma Aldrich) was boiled in a microwave oven and filled as a thin layer into Petri dishes (85 mm diameter). After cooling, closed Petri dishes were kept at room temperature and were used on the same day or on the next day. As putative positive or negative reinforcers, fructose (2 M, FLUKA) or sodium chloride salt (1.5 M, FLUKA), respectively, was added to the agarose solution after boiling. Prior to the experiments, teflon containers [2] were loaded with either pure benzaldehyde (BA), pure 1-octanol (1OCT) or diluted amylacetate (AM, 1∶250 in paraffin oil) as odorant stimuli.

Immediately before the experiment, a small amount of food containing larvae was collected from the food vial and transferred to an empty Petri dish. About 30 larvae were washed with fresh water and placed in the middle of the experimental plates.

All assays were performed in normal light under the fume hood. The lids for the Petri dishes were perforated in the center with tiny holes. Prior to the s*hibire^ts^* experiments, larvae were incubated in their food vials for 30 min on 37°C in a water-bath. The assays were then performed at restrictive temperature in a room heated at about 34°C. All other experiments were done at room temperature of about 23°C. Parts of the experiments were done blind with respect to genotypes. The experimental design of the two-odor assay using AM and BA was the same as previously described [1], [2]. Apart from that, we also used a one-odor paradigm with 1OCT presented together with an empty odor container.

Except for the s*hibire^ts^* experiments, larvae were trained in three cycles, each consisting of five min training with reinforced odor A (CS+) and five min with non-reinforced odor B (CS−). Reciprocal treatment with non-reinforced odor A (CS−) and reinforced odor B (CS+) in another group of larvae was done simultaneously. Immediately after training, larvae were tested for five min for their preference of odor A or odor B. Aversive learning was tested on a salt plate and appetitive learning was tested on a pure plate – as published before [1], [2]. For the assays done at restrictive temperature, a shorter protocol with three training cycles, each 2×2.5 min, was established, in order to minimize unspecific temperature effects on behavior; however, the test session in these experiments also lasted five min. Larvae on each side of the test plate were then counted, and a preference for AM was calculated. Together with the reciprocal procedure, both preference values were used to calculate the performance index.
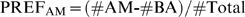


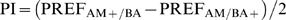
In addition, all genotypes used were also tested for their naïve preferences to the odors and tastants applied. For measuring odor preferences, larvae were tested with either AM, BA, or 1OCT and an empty teflon container on the opposing side. Preference for sugar and avoidance of salt was assayed in a choice test on Petri dishes filled half with pure agarose and half with agarose mixed with the reinforcer solution. After five min, larvae on each side were counted to calculate an olfactory or gustatory preference index, which allowed us to elucidate their perception capacity of these cues.
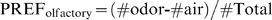


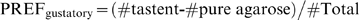



### Statistical Methods

For the comparison between genotypes Wilcoxon Rank Sum test was used. To compare single genotypes against chance level we used the Wilcoxon signed ranked test. All statistical analyses and visualizations were done with R version 2.8.0. Figure alignments were done with Adobe Photoshop. Data were presented as box plots, including all values of a given genotype, 50% of the values being located within the box. The median performance index was indicated as a bold line within the box plot. Significance levels between genotypes shown in the figures refer to the p-value obtained in the statistical tests.

### Immunofluorescence

#### Antibodies

To analyze the expression pattern of TH GAL4, we used a rabbit polyclonal serum against green fluorescent protein (anti-GFP; Molecular Probes, Eugene, OR, 1∶200) and two different mouse antibodies for staining the neuropile (ChAT4B1; DSHB, Iowa City, IA, 1∶150) and the axonal tracts (1d4 anti-Fasciclin II; DSHB, 1∶50), respectively. Overlap of TH expression and the TH-GAL4-pattern was checked via a polyclonal antibody against TH ([92]; 1∶800) and a chicken anti-GFP antibody (Chemicon International, Tenecula, CA, 1∶170). The DA receptors were recognized by a mouse antibody against dDA1 ([41],1∶500) and a polyclonal rabbit antibody against the DAMB receptor (anti-DAMB, [40], 1∶200). Goat anti-rabbit IgG Alexa Fluor 488 (Molecular Probes, 1∶200), fluorescein (FITC)-conjugated donkey anti-chicken (Jackson ImmunoResearch, West Grove, PA, 1∶170), Cy3 goat-anti-mouse IgG (Invitrogen, Eugene, OR, 1∶100) and Cy3 goat anti-rabbit IgG (Jackson ImmunoResearch, 1∶100) were used as secondary antibodies.

### Immunostaining

Third instar larvae were put on ice and dissected in phosphate-buffered saline (PBS). Brains were fixed in 3.6% formaldehyde (Merck, Darmstadt) in PBS for 25 min. After four times washing with PBT (PBS with 3% Triton-X 100, Sigma-Aldrich, St. Louis, MO), brains were blocked with 5% normal goat serum (Vector Laboratories, Burlingame, CA) in PBT for 1.5 hours and then incubated for two days with first antibodies at 4°C. Before applying the secondary antibodies for one day at 4°C, brains were washed six times with PBT. Finally, brains were washed five times with PBT and once with PBS, mounted in Vectashield (Vector Laboratories) between two cover slips and stored at 4°C in darkness. Images were taken with a LeicaTCS SP5 confocal microscope with ×20 or ×63 glycerol objectives. The resulting image stacks were projected and analyzed with Image-J (NIH) software. Contrast and brightness adjustment as well as rotation and organization of images were performed in Photoshop (Adobe Systems Inc., San Jose, CA).

## Supporting Information

Figure S1 Establishing the UAS-*shi^ts1^* protocol. (A) A protocol for testing naïve odor preference of larvae at restrictive temperature. (B) The shading chosen for experimental and control animals. (C) Or83b-GAL4/UAS-*shi^ts1^* larvae did not show any significant preference for benzaldehyde(BA) (p = 0.32) 5 minutes after the preincubation, whereas larvae of both control genotypes were attracted by the odor (p = 0.012 for Or83b-GAL4/+ and p = 0.007 for UAS-*shi^ts1^*/+). (D) Experimental larvae showed no significant preference for amylacetate(AM) (p = 0.565) after 10 min at 34°C, whereas both controls performed over chance level. (E) After 15 min at 34°C, Or83b-GAL4/UAS-*shi^ts1^* larvae still did not perform significantly different from chance level (p = 0.08), whereas Or83b-GAL4/+ and UAS-*shi^ts1^* were still attracted by BA.
(11.96 MB TIF)

Figure S2 Potential Antennal Lobe Innervation by the TH-GAL4 Line. (A–C) Tyrosine hydroxylase (TH) immunoreactivity (green; A) and TH-GAL4/UAS-Cameleon2.1 expressing cells (green; B and C) are shown on fasciclinII (FasII)/cholineacetyltransferase (ChAT) background staining (magenta; A–C). (A and B) Confocal stacks of the anterior part of the cns. (A) The al is not labeled by the TH-antibody (arrow). (B–D) Unilateral innervation of the al by a TH-GAL4 positive cell was observed in one brain (arrow B). Scale bars: A,B 50 µm; C, D 25 µm
(1.50 MB TIF)
