# Supplementary figures and images for: The Role of Dopamine in Drosophila Larval Classical Olfactory Conditioning
Source: PLoS One. 2009 Jun 12;4(6):e5897. doi: 10.1371/journal.pone.0005897 (PMC2690826; doi:10.1371/journal.pone.0005897)

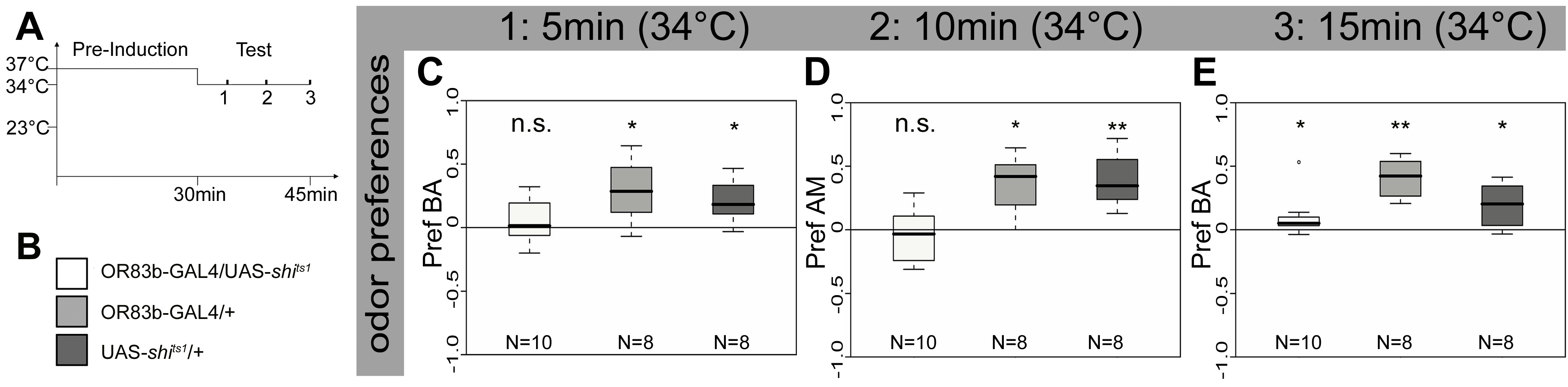

Supplement: Figure S1 — Establishing the UAS-shits1 protocol. (A) A protocol for testing naïve odor preference of larvae at restrictive temperature. (B) The shading chosen for experimental and control animals. (C) Or83b-GAL4/UAS-shits1 larvae did not show any significant preference for benzaldehyde(BA) (p = 0.32) 5 minutes after the preincubation, whereas larvae of both control genotypes were attracted by the odor (p = 0.012 for Or83b-GAL4/+ and p = 0.007 for UAS-shits1/+). (D) Experimental larvae showed no significant preference for amylacetate(AM) (p = 0.565) after 10 min at 34°C, whereas both controls performed over chance level. (E) After 15 min at 34°C, Or83b-GAL4/UAS-shits1 larvae still did not perform significantly different from chance level (p = 0.08), whereas Or83b-GAL4/+ and UAS-shits1 were still attracted by BA. (11.96 MB TIF) [file pone.0005897.s001.tif]

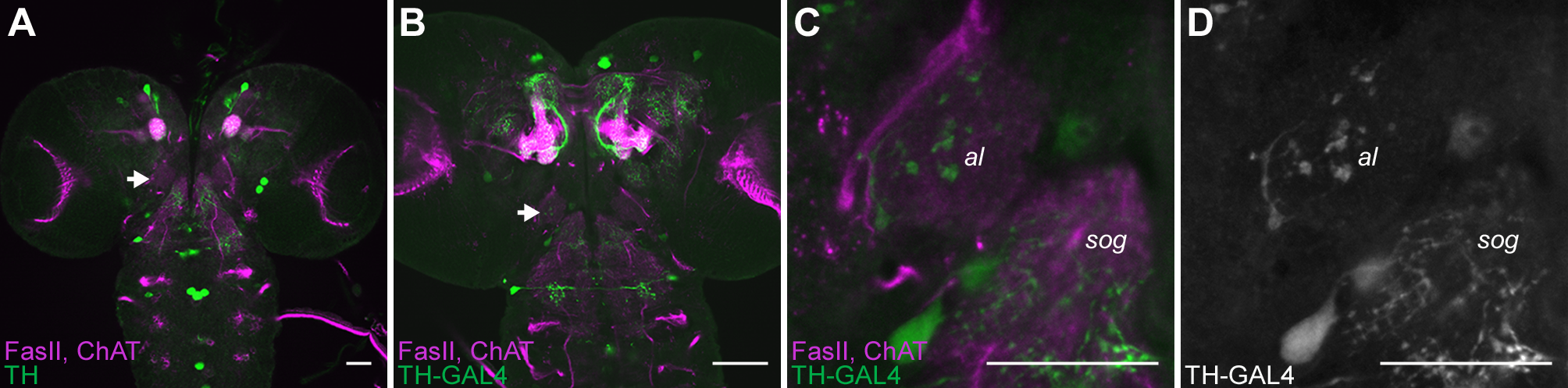

Supplement: Figure S2 — Potential Antennal Lobe Innervation by the TH-GAL4 Line. (A–C) Tyrosine hydroxylase (TH) immunoreactivity (green; A) and TH-GAL4/UAS-Cameleon2.1 expressing cells (green; B and C) are shown on fasciclinII (FasII)/cholineacetyltransferase (ChAT) background staining (magenta; A–C). (A and B) Confocal stacks of the anterior part of the cns. (A) The al is not labeled by the TH-antibody (arrow). (B–D) Unilateral innervation of the al by a TH-GAL4 positive cell was observed in one brain (arrow B). Scale bars: A,B 50 µm; C, D 25 µm (1.50 MB TIF) [file pone.0005897.s002.tif]
